# PEG-coated nanoparticles detachable in acidic microenvironments for the tumor-directed delivery of chemo- and gene therapies for head and neck cancer

**DOI:** 10.7150/thno.45164

**Published:** 2020-05-17

**Authors:** Yu-Li Lo, Chih-Hsien Chang, Chen-Shen Wang, Muh-Hwa Yang, Anya Maan-Yuh Lin, Ci-Jheng Hong, Wei-Hsuan Tseng

**Affiliations:** 1 Institute of Pharmacology, National Yang-Ming University, Taipei 11221, Taiwan.; 2 Faculty of Pharmacy, National Yang-Ming University, Taipei 11221, Taiwan.; 3 Center for Advanced Pharmaceutics and Drug Delivery Research, National Yang-Ming University, Taipei 11221, Taiwan.; 4 Institute of Clinical Medicine, National Yang-Ming University, Taipei 11221, Taiwan.; 5 Division of Medical Oncology, Department of Oncology, Taipei Veterans General Hospital, Taipei 11217, Taiwan.; 6 Department of Medical Research, Taipei Veterans General Hospital, Taipei 11217, Taiwan.

**Keywords:** head and neck cancer, self-cleavable PEG-shell, combinatorial therapy, microRNA, pH-sensitive targeting nanoparticles

## Abstract

**Background:** Head and neck cancer (HNC) is a major cause of morbidity and mortality and has a poor treatment outcome. Irinotecan, a topoisomerase-I inhibitor, induces cell death by decreasing the religation of double-strand DNA. However, epithelial-mesenchymal transition (EMT), therapy resistance, and systemic toxicity caused by available antineoplastic agents hinder the efficacy and safety of HNC treatment. Chemotherapy combined with gene therapy shows potential application in circumventing therapy resistance and EMT. miR-200 exerts a remarkable suppressing effect on EMT-associated genes. Herein, liposomes and solid lipid nanoparticles (SLNs) modified with a pH-sensitive, self-destructive polyethylene glycol (PEG) shell and different peptides were designed as irinotecan and miR-200 nanovectors to enhance tumor-specific accumulation. These peptides included one ligand targeting the angiogenic tumor neovasculature, one mitochondrion-directed apoptosis-inducing peptide, and one cell-penetrating peptide (CPP) with high potency and selectivity toward cancer cells.

**Methods:** Physicochemical characterization, cytotoxicity analysis, cellular uptake, regulation mechanisms, and *in vivo* studies on miR-200- and irinotecan-incorporated nanoparticles were performed to identify the potential antitumor efficacy and biosafety issues involved in HNC treatment and to elucidate the underlying signaling pathways.

**Results:** We found that the cleavable PEG layer responded to low extracellular pH, and that the CPP and targeting peptides were exposed to improve the uptake and release of miR-200 and irinotecan into HNC human tongue squamous carcinoma (SAS) cells. The apoptosis of SAS cells treated with the combinatorial therapy was significantly induced by regulating various pathways, such as the Wnt/β-catenin, MDR, and EMT pathways. The therapeutic efficacy and safety of the proposed co-treatment outperformed the commercially available Onivyde and other formulations used in a SAS tumor-bearing mouse model in this study.

**Conclusion:** Chemotherapy and gene therapy co-treatment involving pH-sensitive and targeting peptide-modified nanoparticles may be an innovative strategy for HNC treatment.

## Introduction

Head and neck cancer (HNC) is one of the most significant causes of morbidity and mortality globally and has a poor treatment outcome 1. Among HNCs, oral squamous cell carcinoma (OSCC), especially of the tongue, is one of the most diagnosed oral cancer phenotype 2. Human tongue squamous carcinoma (SAS) cells are notably highly aggressive, migratory, and invasive 3. Thus, SAS cells were used in the present study to represent a tumor-progression model of OSCC 4. Current HNC chemotherapeutic agents such as 5-fluorouracil and cisplatin have been considered standard treatments, but OSCC is frequently unresponsive to common chemotherapy and is usually accompanied by early relapse, distant metastasis, and poor prognosis 5. Irinotecan (Iri), a camptothecin derivative, is a validated option in second- and third-line settings 6. However, therapy resistance and unremarkable improvements have been observed in currently available chemotherapeutics 7. Epithelial-to-mesenchymal transition (EMT), resistance to current chemotherapy, and systemic toxicity caused by available antineoplastic agents hinder the efficacy and safety of HNC treatment 8. Iri, a topoisomerase (Topo)-I inhibitor, induces cell death by inhibiting the religation of double-strand DNA 9. Iri and its metabolite SN-38 are typically pumped outside cancer cells by P-glycoprotein (P-gp) and multidrug resistance (MDR)-associated proteins (MRPs), leading to MDR 10. EMT also contributes to the development of acquired Iri resistance and elevated migration and invasion in different cancer types 11,12. However, the capacity of current HNC therapies to suppress MDR and EMT is limited 13. Accordingly, identifying suitable gene therapeutics for co-treatment with Iri is urgent to effectively inhibit Iri resistance and increase the chemosensitivity of HNC to this drug.

MicroRNAs (miR), small noncoding RNAs of 18-25 base pairs, modulate mRNA expression at the post-transcriptional level 14. EMT activation plays a critical role in tumor invasion and metastasis 15. Thus, repressing EMT action as a potential anticancer strategy is very important. The overexpression of zinc finger E-box binding homeobox 1 (ZEB1) triggers EMT by suppressing miR-200 family members 16,17. Accordingly, miR-200 family upregulation is critical to inhibit the expression levels of ZEB1 and ZEB2, thereby reducing EMT in OSCC 18. Furthermore, the induction of the miR-200c-3p expression inhibits ZEB1 function and increases the sensitivity to target therapy (i.e., MEK inhibition) in KRAS mutation-resistant lung cancer 19. Moreover, the downregulation of miR-200 family members plays a key role in the anti-apoptosis, progression, invasion, metastasis, and drug resistance of different cancers (e.g., OSCC, cervical cancer, pancreatic cancer, nasopharyngeal carcinoma, and soft-tissue sarcomas) 20. Nevertheless, the use of miR-200 alone ineffectively suppresses tumors 16. Thus, the administration of miR-200 in solid lipid nanoparticles (SLNs) and Iri in liposomes (Lip) may improve current clinical problems such as rapid degradation, limited tumor penetration, and low uptake into cancer cells, which are associated with chemo- and gene therapies 21. The pretreatment of anionic miR-200 in cationic SLNs reportedly activates related signaling pathways such as EMT suppression to increase the cytotoxic potency of using Iri-loaded anionic Lip against colorectal cancer (CRC) in HCT116 cells and CRC-bearing mice 22.

SLN and Lip, which are decorated with cell-penetrating peptides (CPPs), show different characteristics in reducing nonselective cellular uptake and enhancing specific tumor targeting. CPPs are a group of short cationic peptides usually rich in arginine and lysine 23. **N peptide** can bind to nerve/glial antigen 2 (NG2) overexpressed in the tumor neovasculature and thus functions as a targeting ligand 24. Given the selectivity of the N peptide in tumor cells, it may serve as a prospective modification for the tumor-specific delivery of nanomedicines 24. N peptide modification reportedly enhances nanoparticle internalization via the binding of N peptide to NG2 receptor, leading to effective antiangiogenic therapy 25. **C peptide** displays high potency and selectivity against cancer cells, but it shows lower toxicity to normal cells than that of TAT peptide 26. Interestingly, our pervious study has also indicated that the transport of gefitinib-encapsulated Lip modified with C peptide across the blood-brain barrier is improved by modulating the transcytosis pathway primarily via the adsorptive-mediated mechanism 27.** M peptide**, a mitochondrion-localizing peptide, possesses pro-apoptotic KLA residues 28. This peptide potentiates mitochondrion-mediated lung carcinoma cell death via membrane-potential reduction and ATP depletion 29. M peptide-conjugated nanostructures also facilitate doxorubicin accumulation in mitochondria, thereby triggering the release of cytochrome c and increasing the expressions of caspase-9, caspase-3, p21, and p53 30.

**Figure 1:**
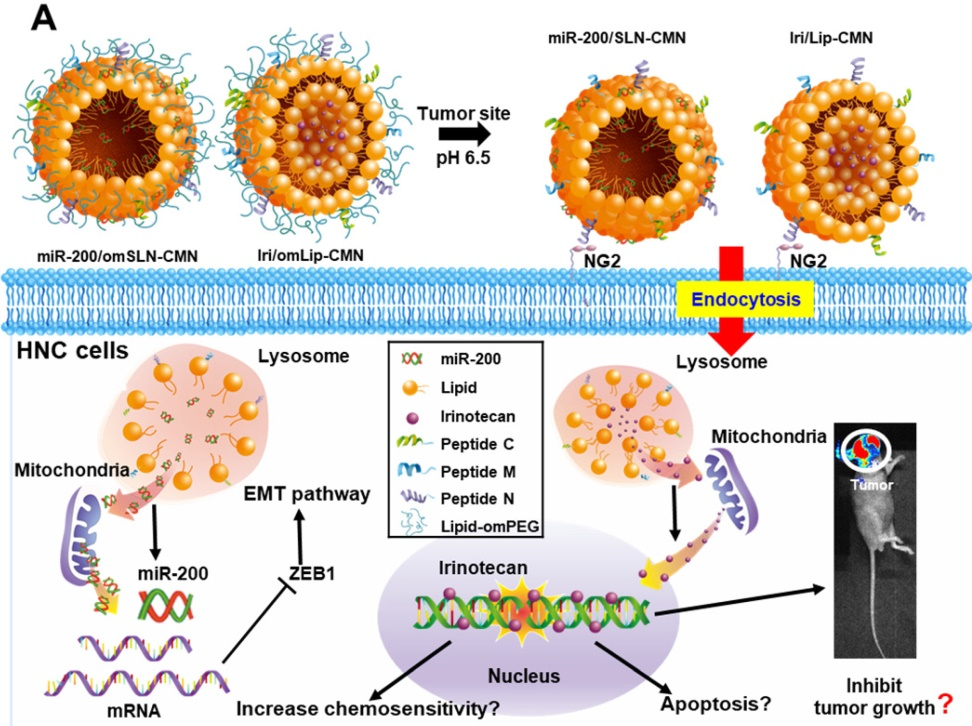

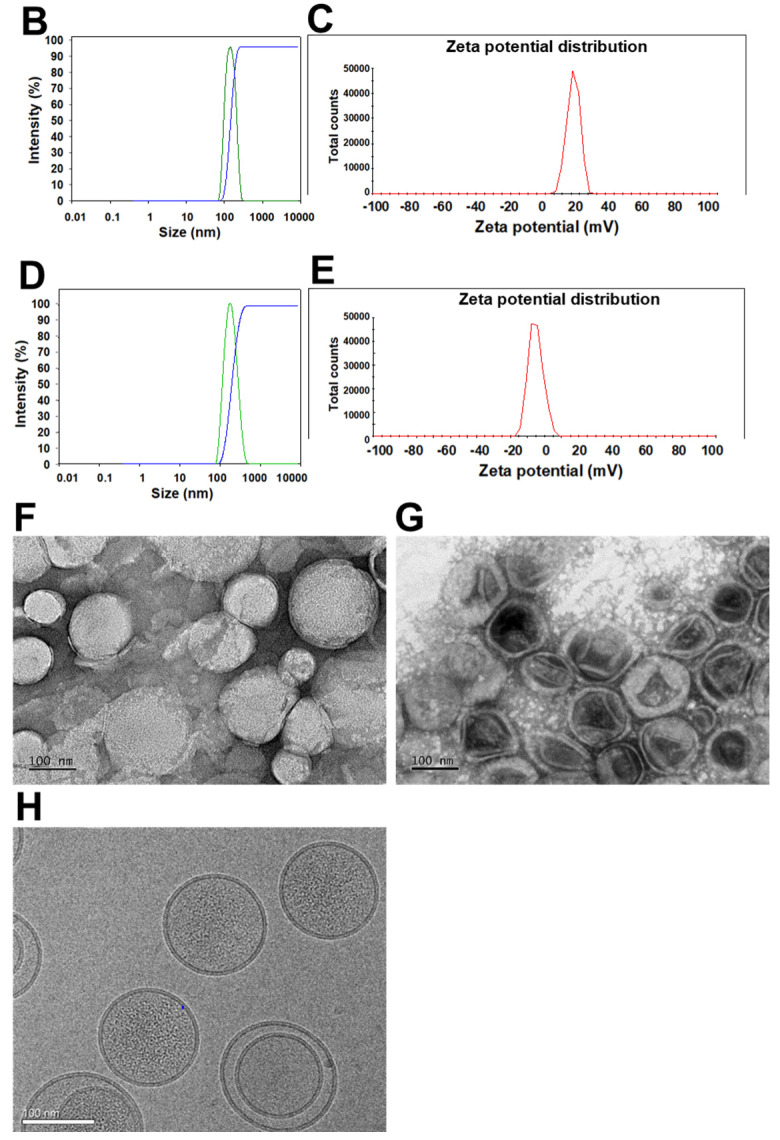
(A) Scheme of the rationale of pH-sensitive and peptide-modified liposomes (Lip) and solid lipid nanoparticles (SLN) incorporating irinotecan (Iri) and miR-200, respectively. (B-G) Sizes and zeta potential of (B-C) omSLN-CMN and (D-E) omLip-CMN were measured with Malvern Zetasizer. TEM images of (F) miR-200/omSLN-CMN and (G) Iri/omLip-CMN were observed using JEM-2000EXII TEM. (H) Cryo-TEM images of Iri/omLip-CMN. Bar = 100 nm. For each group, *n* = 3.

In the present study, the lipid core of SLNs and Lip is modified by N peptide for tumor targeting, M peptide for mitochondrion directing, and C peptide for enhanced cancer penetration to prepare **SLN-CMN** and **Lip-CMN**. Moreover, the PEG derivative Oʹ-methyl polyethylene glycol (omPEG) was conjugated into lipid to form a pH-sensitive imine bond. SLN-CMN and Lip-CMN were then additionally modified with this cleavable long-chain PEG-lipid derivative (lipid-imine-omPEG) to prepare **omSLN-CMN** and **omLip-CMN** (Figure 1A). This design of two nanoparticles coated with multifunctional peptides and a PEG derivative responsive to an acidic tumor environment may provide potential basis for the separate delivery of combinatorial therapy comprising Iri and miR-200. This strategy may increase passive tumor targeting via the enhanced permeability and retention (EPR) effect, intensify active tumor targeting through specific ligand-receptor binding, and improve endosomal escape and mitochondrial localization.

## Materials and methods

### Materials

FAM-miR-200 and has-miR-200c-3p were synthesized by GenePharma (Shanghai, China). Iri hydrochloride was purchased from AK Scientific (Union City, CA, USA). C, M, and N peptides were custom synthesized by Kelowna (Taiwan). Cholesterol and paraformaldehyde were bought from Acros (Geel, Antwerp, Belgium). All lipids were obtained from Avanti (Alabaster, AL, USA). Lipofectamine™ 3000 was procured from Thermo Fisher Scientific (Waltham, MA, USA). All cell culture media and reagents were bought from Gibco BRL (Grand Island, NY, USA). Most of the other chemical reagents were obtained from either Merck (Darmstadt, Germany) or Sigma-Aldrich (St. Louis, MO, USA).

### Synthesis of 1,2-distearoyl-sn-glycero-3-phosphoethanolamine-omPEG

1,2-distearoyl-sn-glycero-3-phosphoethanolamine (DSPE) was dispersed in chloroform/methanol (9:1), and omPEG was added to the solution. The mole ratio of DSPE and omPEG was 1:1. The mixture was allowed to react overnight at 50 °C. DSPE-omPEG was then obtained after the organic solvent was removed using a centrifugal evaporator (Genevac SF50, Genevac Ltd., Ipswich, England, UK). DSPE-omPEG was examined with ^1^H NMR (400 MHz, Bruker Avance III, Rheinstetten, Germany) to confirm the structure of the conjugate.

### Synthesis of peptide-conjugated lipid

DSPE-PEG-maleimide was dissolved in chloroform/methanol (9:1). C, M, and N peptides were added to the lipid solution (individual peptide/lipid molar ratio = 1:1) and allowed to react overnight. After evaporation, the residue was dissolved in water and dialyzed against water overnight to remove unconjugated peptides by using a dialysis bag (3.5-5 kDa membrane; Spectrum Laboratories, CA, USA). The final product (DSPE-PEG-peptide) was lyophilized, and the structure was verified through matrix-assisted laser desorption ionization time-of-flight mass spectrometry (MALDI-TOF MS; Applied Biosystems, MA, USA).

### Preparation of peptide-conjugated and pH-sensitive Iri/omLip-CMN

Peptide-conjugated and Iri-loaded Lip (Iri/Lip-CMN) were prepared by thin-film hydration. The molar ratio of DSPC, cholesterol, DSPE-PEG-peptide, and DSPE-omPEG was 1:0.1:0.1:0.1. In a typical procedure, the above mixture at the indicated ratio was dissolved in chloroform/methanol (9:1). After the organic solvent was removed, the lipid thin film was suspended in phosphate-buffered saline (PBS) at 37 °C. The Lip were extruded through 400, 200, and 100 nm membrane filters. Iri was then added to blank Lip, and further incubation was performed at 50 °C for 1 h through an ammonium sulfate gradient method to obtain Iri-loaded Lip.

### Preparation of peptide-conjugated and pH-sensitive miR/om SLN-CMN

SLN were prepared by dispersing L-α-phosphatidylcholine (PC), cholesterol, DOTAP, DSPE-PEG-peptide, and DSPE-omPEG at a molar ratio of 1:0.1:0.1:0.1:0.1 in methanol/dichloromethane. The above mixture was added dropwise into Tween 80 solution. A miR solution was loaded into the SLNs, and the final dispersion was maintained at room temperature for 30 min.

### Characterization of various Lip and SLN formulations

The size distribution and zeta potential of Iri/omLip-CMN and miR/om SLN-CMN were determined using a Zetasizer Nano-ZS particle-size analyzer (Malvern Instruments Ltd., Malvern, Worcestershire, England, UK). The morphological characteristics of these formulations were imaged under a transmission electron microscopy (TEM) system (JEM-2000EXⅡ, Japan). Morphology was further visualized using a cryo-TEM instrument (Tecnai G2 F20 TWIN, FEI Company, The Netherlands).

### Encapsulation efficiency (EE%) and drug-loading capacity (DL%)

A dispersion of Iri- or miR-incorporated nanoparticles was centrifuged at 15 000 rpm and 4 °C by using an ultracentrifuge filter (Amicon®) for 30 min. The filtrate was collected and analyzed with an Ultrospec 8000 PC spectrophotometer (Biochrom Ltd., Cambridge, England, UK) and NanoDrop (Thermo Fisher, MA, USA). The collected nanoparticles were broken with 0.5% Triton X 100, and the residual nanoparticles were dissolved in methanol and chloroform after centrifuging at 15 000 rpm and 4 °C for 30 min. Iri or miR was analyzed as mentioned above. Each sample was detected in triplicate. The EE% and DL% of Iri or miR in different formulations were computed using the following formulas:

EE% = [(W_1_ - W_2_)/W_1_]×100% (1)

DL% = [(W_1_ - W_2_)/W_3_]×100% (2)

where W_1_ is the weight of the added Iri or miR, W_2_ is the weight of Iri or miR in the filtrate, and W_3_ is the weight of the total phospholipids.

### Protection test of miR-loaded formulations via gel-retardation assay

miR was encapsulated with or without different SLN formulations (SLN, SLN-CMN, and omSLN-CMN) and incubated with 1% RNase or 50% fetal bovine serum (FBS) at 37 °C. The samples were loaded into the gel, run with polyacrylamide gel (PAGE) at 60 V, and stained with ethidium bromide at 25 °C for 30 min. Afterwards, the gel was monitored and scanned using a gel-documentation system (DigiGel, TopBio, Taipei, Taiwan).

### Changes in pH-induced size and zeta potential

Changes in the pH-sensitive size and zeta potential of Lip-CMN and SLN-CMN with or without pH-sensitive omPEG or PEG layer (no imine bond for comparison) were detected using Malvern Zetasizer Nano ZS90. These nanoparticles were incubated in PBS (pH 7.4 and 5.5) at 25 °C for 1 h. The size and zeta potential of each sample were measured in triplicate.

### In vitro pH-sensitive release

To verify the pH-sensitive drug release, different Iri- or miR-encapsulated preparations were maintained in a dialysis bag (1000-3500 MWCO) and dialyzed sequentially against PBS at pH 7.4 and 6.5 at 37 °C. At the designated time (0, 1, 2, 5, 8, 12, 24, 48, and 72 h), samples were withdrawn from the medium and substituted with an equal volume of fresh medium. Iri- or miR concentrations were detected using a spectrophotometer as mentioned above to calculate the cumulative drug-release percentage.

### Cell lines

SAS and SAS/luc cells, obtained from Professor Muh-Hwa Yang's lab, were cultured in Dulbecco's modified Eagle's medium (DMEM) supplemented with 10% FBS. Normal oral keratinocyte (NOK) cells were cultured in DMEM with 10% FBS, 100 IU/mL penicillin, and 100 mg/mL streptomycin.

### pH-sensitive cellular uptake

The cellular uptake of SAS and NOK cells was quantified by flow cytometry. The cells were treated with various formulations incorporating daunorubicin (DNR; a fluorescent probe for Iri), and the collected cell pellets were washed with 1 mL of cold PBS and re-suspended in PBS (pH 7.4) for NOK and PBS (pH 7.4 and 6.5) for SAS. The fluorescence intensity of DNR uptaken by the cells was measured with a FACSCalibur flow cytometer (BD Biosciences, San Jose, CA, USA).

### Transfection study

Various FAM-miR-200 formulations were added to SAS cells, and the mixtures were incubated for 24 h. The cell pellets were centrifuged, collected, and washed with 1 mL of cold PBS. The fluorescence intensity of FAM-miR-200 that entered the cells was detected through flow cytometry and compared with that of commercially available transfection agents, such as T-Pro, Viromer, and Lipofectamine 3000.

### Identification of cellular-uptake pathways

SAS cells were preincubated with different endocytosis inhibitors at 37 °C for 1 h. The endocytosis inhibitors included 5-(N,N-dimethyl) amiloride (DMA), nystatin, chlopromazine (CPZ), poly-L-lysine, and methyl-β-cyclodextrin (MBCD). After incubating the cells with DNR/Lip-CMN and FAM-miR/SLN-CMN for another 3 h, the resulting cell pellets were harvested and washed with cold PBS. Fluorescence intensity was then determined using a flow cytometer.

### Identification of intracellular localization

SAS cells were seeded, incubated with DNR/Lip-CMN or FAM-miR/SLN-CMN, fixed with 4% paraformaldehyde for 10 min, and stained with MitoTracker® Green (MitoGreen) or MitoTracker® Red (MitoRed) to monitor mitochondrial localization. NG2 was identified by immunofluorescence staining with cyan-labeled anti-NG2 antibody overnight. The cells were stained with DAPI at 37 °C to locate the nucleus. Images were taken using a confocal laser scanning microscopy (CLSM) instrument (Olympus FV10i).

### Cell-viability evaluation by sulforhodamine B (SRB) assay

NOK or SAS cells were seeded in 96-well plates overnight. The cells were pretreated with miR-200 formulations for 24 h and then treated with various Iri formulations for 24 h at 37 °C. Cell viability was measured by SRB assay. Afterwards, 0.04% SRB was added to the individual well for 10 min, and each well was washed thrice with 1% acetic acid. The wells were dried at room temperature for 24 h, and then 10 mM Tris base was added to each well. Absorbance was detected at 540 nm by using a microplate TECAN reader.

### Annexin V-propidium iodide (PI) assay

The cells were treated with six formulations, namely, control (CTR), Iri, Iri/Lip, Iri/Lip-CMN, miR-200/SLN-CMN, and miR-200/SLN-CMN+Iri/Lip-CMN, for 48 h. To determine the percentage of cell populations in apoptosis, necrosis, and death, the harvested cells were washed and stained with Annexin V-PI labeling solution in darkness. The apoptosis, necrosis, and death (%) of SAS cells were monitored and computed using a BD flow cytometer.

### Western blot assay

SAS cells were seeded, incubated with the above-mentioned six formulations for 24 h, and lysed through radioimmunoprecipitation assay. Proteins were examined by BCA protein assay. Protein samples were separated by SDS-PAGE and transferred onto polyvinylidene difluoride membranes. After blocking the membranes via nonspecific binding with 5% nonfat milk for 1 h, blots were incubated with primary antibodies against various proteins from Cell Signaling or Abcam at 4 °C overnight and conjugated with horseradish peroxidase-linked immunoglobulin G (Jackson). After being developed in a Millipore detection system and reprobed with anti-β-actin antibody, these blots were visualized with enhanced chemiluminescence kits (PerkinElmer).

### Migration assay

SAS cells were seeded in inserts (Ibidi GmbH, Munich, Germany), treated with the different formulations, and monitored for 15 h. Images were obtained through optical microscopy, and the migration area was quantified using Image J. Relative migration percentages were calculated using the following equation:

Relative migration area (% of area at 0 h) = 100% - [blank area_(15h)_/blank area_(0h)_ × 100%]. (3)

### Establishment of *in vivo* SAS-tumor bearing mouse model

Male BALB/c nude (nu/nu) mice (6 weeks old; ~22 g body weight) were purchased from the National Laboratory Animal Center and maintained in the Animal Center of National Yang-Ming University. Animal care and handling procedures were in accordance with the guidelines approved by the Institutional Animal Care and Use Committee. SAS-luc cells were injected subcutaneously into the right cheek region of the mice. Tumor size was calculated as follows:

V = (L × W^2^)/2 (4)

where L is the longest diameter (mm), and W is the shortest diameter (mm) perpendicular to the longest axis.

### Antitumor efficacy, body weight, and IVIS imaging evaluation of SAS tumor-bearing mice

Tumors were allowed to grow to approximately 60 mm^3^ before treatment. SAS/luc tumor-bearing mice were randomly divided into eight groups (n = 5; Iri = 40 mg/kg; miR-200 = 1.25 mg/kg): 1) saline (control, CTR); 2) Iri; 3) Iri/Lip; 4) Iri/Lip-CMN; 5) miR-200/SLN-CMN + Iri/Lip-CMN; 6) Iri/omLip-CMN, miR-200/omSLN-CMN + Iri/omLip-CMN; 7) Onivyde; and 8) miR-200/omSLN-CMN + Onivyde 22,31. For the combined treatment of miR-200 and Iri formulations, after treatment with miR-200/SLN-CMN for 24 h, various Iri formulations were administered. Each group received different formulations on the 1st and 14th days. The tumor size and body weight of the mice were measured with a digital caliper every 2 days after treatments, and tumor volume (V) was computed according to Equation 4. Fluorescence images were visualized with IVIS Spectrum (PerkinElmer, Waltham, MA, USA) 1 day after the final treatment. Survival percentage was calculated as the survival number of mice at the indicated time divided by the initial number of mice.

### Biodistribution, biochemical tests, and hematoxylin and eosin (H&E) staining

After 48 h of the final treatment, 170 μL of blood samples was obtained from the orbital sinus of mice. After centrifugation, the serum levels of glutamate pyruvate transaminase (GPT), creatinine (CRE), and creatine kinase-MB (CK-MB) were detected using the corresponding activity assay kits (Fujifilm, Tokyo, Japan) in a clinical dry-chemistry analyzer (Fuji Dri-Chem 7000 V, Fujifilm Corp.) to evaluate liver, kidney, and heart functions.

After the mice were sacrificed, their liver, kidney, intestine, and tumor tissues were preserved in 4% formaldehyde and embedded in paraffin for H&E staining. The tissue samples were collected, frozen rapidly in liquid nitrogen, and stored at -80 °C. A tissue size of approximately 100 mg was transferred into a glass vial. The tissues were disrupted by manual grinding with a mortar and pestle. After adding methanol and water to extract Iri from the tissues, the samples were vortexed and centrifuged at 3000 rpm and 4 °C. The upper layer of each sample was transferred into separate glass vials, and the amount of Iri was analyzed with an Ultrospec 8000 PC spectrophotometer (Biochrom Ltd., Cambridge, England, UK).

### Terminal deoxynucleotidyl transferase dUTP nick end labeling assay (TUNEL)

TUNEL assay was carried out to examine *in vivo* apoptosis induction in the tumor tissues and vessels of SAS-bearing mice. In a typical procedure, tumor and vessel samples were collected, frozen, and fixed with 4% paraformaldehyde for 20 min. The samples were mixed with a solution of an in situ cell death detection kit (Roche, Germany) in accordance with the manufacturer's instruction. Afterwards, the final products were stained with nuclear Hoechst dye for comparison and observed by CLSM.

### Statistical analysis

Experimental data were expressed as the mean ± standard deviation. Statistical significance was analyzed with Student's t-test to compare the differences between the two treatment groups. Statistical analysis was also carried out using one-way ANOVA and Dunnett's multiple comparison tests. Differences at *P < 0.05, **P < 0.01, and ***P < 0.001 were considered statistically significant.

## Results and discussion

### Characterization of miR-200/omSLN-CMN and Iri/omLip-CMN

The imine bond of DSPE-omPEG successfully formed as confirmed by the peak at 9.35 ppm in the ^1^H NMR spectra (Figure S1A). The conjugation of N, M, and C peptides with DSPE-PEG was verified through MALDI-TOF MS (Figures S1B-E).

The size, zeta potential, polydispersity index (PDI), EE%, and DL% of SLN and Lip with or without omPEG and CMN modification are summarized in Table 1. The mean diameters of miR-200/omSLN-CMN and Iri/omLip-CMN were 148.6 ± 0.36 and 175.2 ± 2.27 nm (Figures 1B-D), respectively, with a PDI of approximately 0.1-0.2. The zeta potentials of miR-200/omSLN-CMN and Iri/omLip-CMN were 16.70 ± 1.69 and -8.18 ± 1.43 mV (Figures 1C-E), respectively. Table 1 also shows the high EE% (>85%) and acceptable DL% of omLip-CMN and omSLN-CMN. Meanwhile, TEM and cryo-TEM were performed to investigate the morphological characteristics of miR-200/omSLN-CMN and Iri/omLip-CMN, and the results are shown in Figures 1F-H. The cryo-TEM image revealed that the om-PEG shell was coated around the Iri-loaded nanoparticle formulation, displaying the traditional bilayer structure of Lip with enclosed Iri crystals in the core (Figure 1H). Moreover, the miR protective features of different nanoparticle formulations were assessed. Results showed that omSLN-CMN completely prevented miR from degradation under the extreme condition of 50% FBS (Figure S2A). This finding suggested the necessity of this pH-sensitive imine bond in enhancing miR-protection ability. omSLN-CMN may have compacted miR into a condensed structure and thus prevented its degradation by nuclease. Additionally, more than 97% of Iri or miR were released from Iri or miR solution within the first 1 h, and the release percentage approached 100% within 12 h as indicated by the 72 h release profiles of Iri or miR at 37 °C (Figures S3A-B). Nevertheless, the percentages of Iri and miR released from omLip-CMN and omSLN-CMN at pH 7.4 were 55.85% ± 1.75% and 58.52% ± 1.99%, respectively, up to 72 h (Figures S3A-B), thereby verifying the limited release property of omLip-CMN and omSLN-CMN at physiological pH. Remarkably, at pH 6.5, the release percentage of Iri and miR from omLip-CMN and omSLN-CMN increased to 75.75% ± 2.11% and 80.29% ± 1.87%, respectively, at 72 h (Figures S3A-B). This finding confirmed the pH-responsive release characteristics of omLip-CMN and omSLN-CMN in acidic pH.

### pH-sensitive characteristics of omLip-CMN and omSLN-CMN at pH 7.4 and 6.5

**Figure 2:**
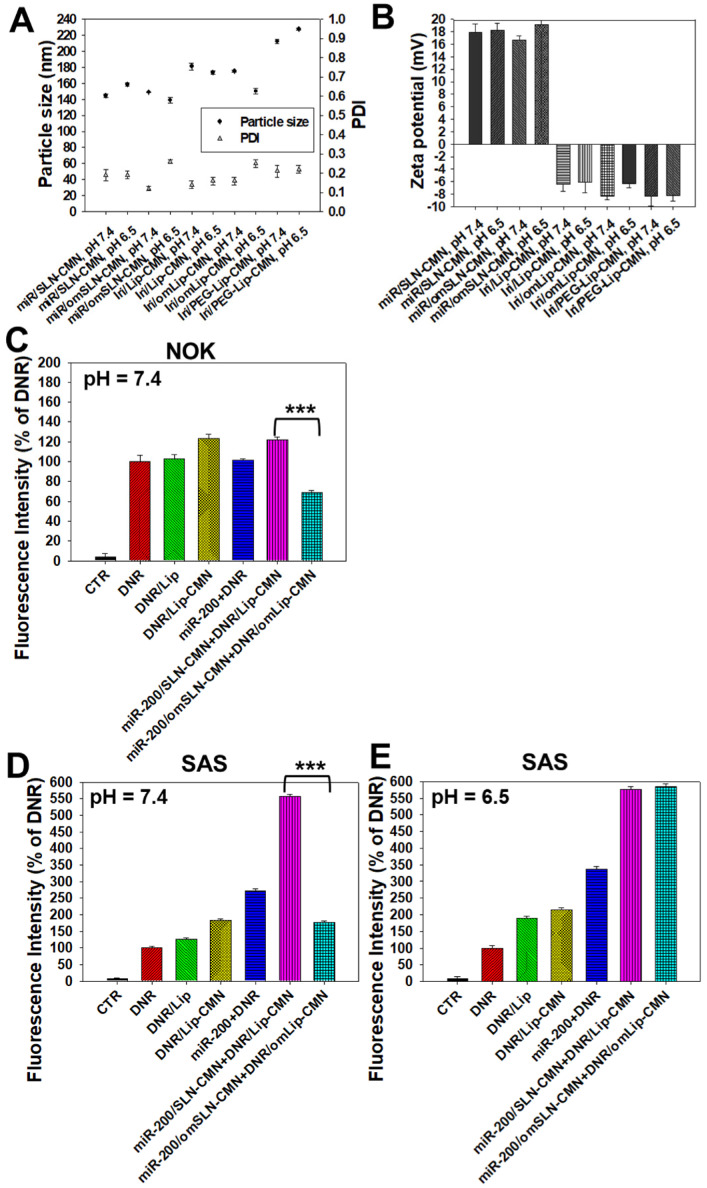
Changes in the pH-sensitive particle size, zeta potential, and cellular uptake of miR-200 and/or Iri formulations at pH 7.4 and 6.5. Changes in (A) sizes, PDI, and (B) zeta potential of miR-200 and/or Iri formulations were measured using a Zetasizer at 30 min after incubation with PBS at pH 7.4 or 6.5. (C-E) Cellular uptake of daunorubicin (DNR; a probe of irinotecan) in various formulations into (C) NOK cells at pH 7.4 and SAS cells at (D) pH 7.4 and (E) 6.5 for 24 h, as detected by flow cytometry.

The pH-responsive changes in the size, PDI, and zeta potential of miR-200/omSLN-CMN and Iri/omLip-CMN at pH 7.4 and 6.5 are displayed in Figures 2A-B and S2B-E. Interestingly, after miR-200/omSLN-CMN were incubated with PBS (pH 6.5) for 30 min, another peak of the de-shedded omPEG layer with large particle size (Figure S2B). After de-coating, the zeta potential of the major peak increased to 18.79 ± 8.12 mV, with a smaller separate peak at approximately -5 mV (Figures 2B and S2C). The particle sizes and zeta potentials of Iri/omLip-CMN at pH 7.4 and 6.5 also exhibited similar trends (Figures 2A-B and S2D-E). These findings revealed the removal of the DSPE-omPEG layer in the acidic tumor microenvironment to expose the cationic peptide-modified SLN-CMN and the less negatively charged Lip-CMN (compared with omLip-CMN). For comparison, we also prepared nanoparticles using DSPE-PEG5000 without an imine bond (Iri/PEG-Lip-CMN; the last two items in Figures 2A-B). The zeta potential of Iri/PEG-Lip-CMN at both pH 6.5 and 7.4 were similar to that of Iri/omLip-CMN at pH 7.4. By contrast, Iri/PEG-Lip-CMN at pH 6.5 showed slightly larger particle sizes than did Iri/PEG-Lip-CMN at pH 7.4, suggesting the absence of pH-dependent de-shielding of the PEG layer for Iri/PEG-Lip-CMN (Figures 2A-B).

The effects of different pH conditions on the uptake of DNR (as a probe of Iri) incorporated into different formulations were examined in noncancerous NOK cells and HNC SAS cells through flow cytometry. Results showed that the fluorescence intensities of DNR in NOK (Figure 2C) and SAS (Figure 2D) cells delivered by omLip-CMN were much lower than those delivered by Lip-CMN at pH 7.4, suggesting the excellent prevention of chemotherapeutics from being uptaken into normal and cancer cells by the om-PEG layer coating. These results also indicated the low intracellular accumulation of anticancer drugs delivered by omLip-CMN at physiological pH. However, the hydrolysis of the imine bond in omLip-CMN at pH 6.5 caused the exposure of the CMN-peptides modified on the surface of Lip-CMN, which remarkably increased the cellular uptake of DNR at pH 6.5 (Figure 2E). This result suggested that omLip-CMN may have prevented the entrance of the chemotherapeutic agent into normal cells (pH 7.4) and enhanced their pH-responsive uptake in the acidic tumor site (pH 6.5). This finding was consistent with our previous study using pH-sensitive nanoparticles 22. Li et al. also reported the design of pH-sensitive boronate esters to release the chemotherapeutic agent bortezomib in a covalently assembled dopamine nanocarrier 32. Fan et al. further used a pH-sensitive PEG shell de-coated from the inner core at the tumor site; they found increased cellular accumulation and fast release of fluorescence probe within the tumor cells, as well as enhanced photodynamic-therapy efficacy 33.

### Mechanisms of cellular internalization and endosomal escape

**Figure 3:**
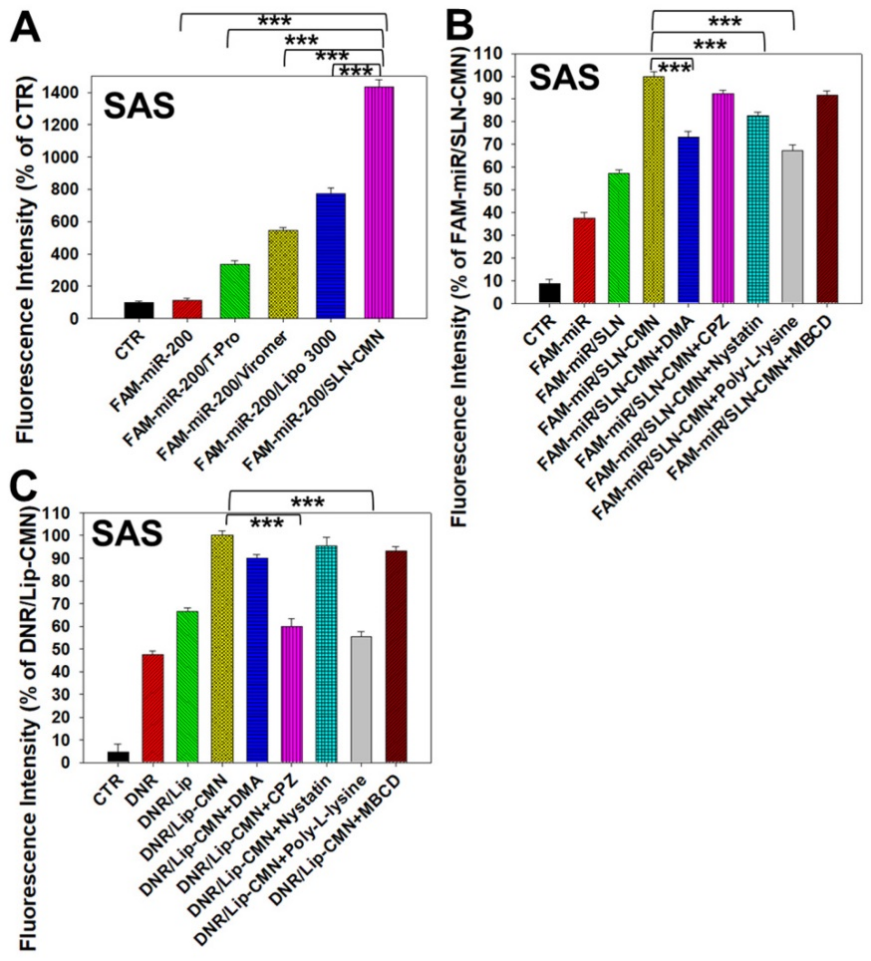

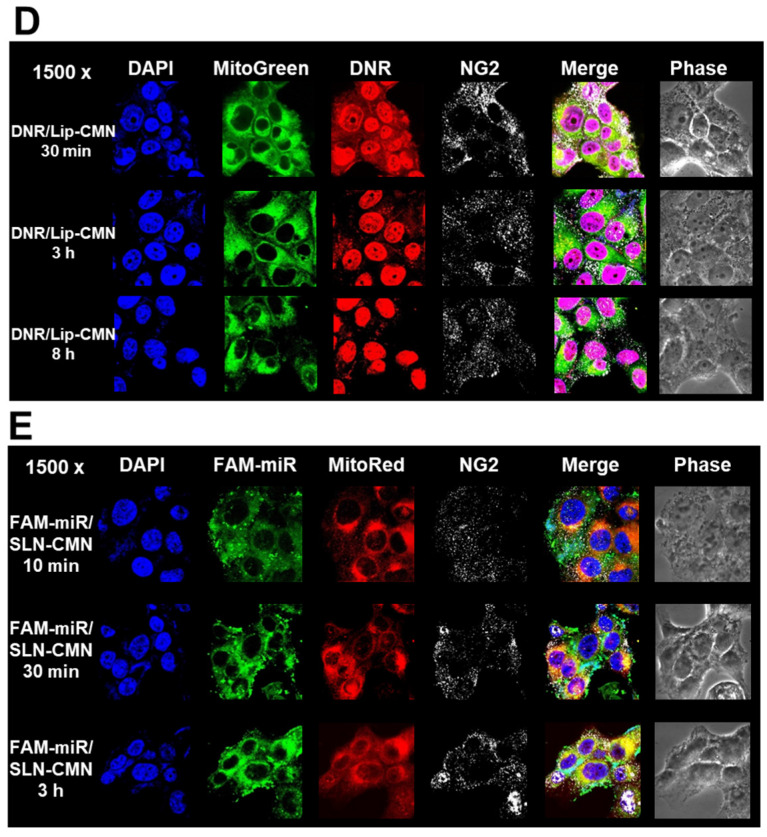
Transfection, cellular internalization, and intracellular trafficking of various miR-200 formulations in SAS cells. (A) Flow cytometry of the transfection efficiency (%) of FAM-miR-200 in the presence of different transfection reagents for 24 h. FAM-miR-200/Lipo 3000: FAM-miR-200/Lipofectamine 3000. (B) Endocytosis mechanisms of FAM-miR-200/SLN-CMN in SAS cells were detected by incubating the cells with specific endocytosis inhibitors at 37 °C for 1 h. Then, the cells were treated with FAM-miR formulations for another 3 h. The endocytosis inhibitors included 5-(N,N-dimethyl) amiloride (DMA), nystatin, chlopromazine (CPZ), poly-L-lysine, and methyl-β-cyclodextrin (MBCD). ***P < 0.001 compared with FAM-miR-200/SLN-CMN without the inhibitor. (C) After treatment with the above endocytosis inhibitors for 1 h at 37 °C, the cells were treated with different DNR formulations for another 3 h. ***P < 0.001 compared with DNR/Lip-CMN without the inhibitor. (D) DNR/Lip-CMN was added to the cells for 30 min, 3 h, and 8 h. Intracellular localization in SAS cells was observed through CLSM. Blue: DAPI (a nuclear dye); green: MitoGreen (MitoTracker Green; a mitochondrial dye); red: DNR; Gray: NG2 (nerve/glial antigen 2). (E) FAM-miR-200/SLN-CMN was added to the cells for 10 min, 30 min, and 3 h. Intracellular trafficking was observed by CLSM in SAS cells. Blue: DAPI; green: FAM-miR200; red: MitoRed (MitoTracker Red; a mitochondrial dye); gray: NG2.

Naked miR has short circulation time and limited intracellular accumulation at tumor sites because of rapid degradation by nucleases and poor endosomal escape 34. Thus, we developed SLN-CMN to deliver miR-200 in SAS cells. As shown in Figure 3A, the relative transfection percentage of FAM-miR-200 by SLN-CMN was higher than that of common transfection reagents, including T-pro, Viromer® BLUE, and Lipofectamine™ 3000. This finding revealed the superior transfection enhancement of SLN-CMN primarily owing to the characteristic SLN design and specific modification of SLN by ligand N, CPP C, and mitochondrion-directing peptide M.

Nanoparticles are transported across the cell membrane via endocytic pathways 35, such as macropinocytosis and clathrin- and caveolae-dependent endocytosis 36. Thus, we used different endocytosis inhibitors to block the internalization pathways in the present study. The cellular internalization inhibitors included DMA (macropinocytosis inhibitor), nystatin (caveolae-mediated endocytosis inhibitor), CPZ (clathrin-mediated endocytosis inhibitor), poly-L-lysine (adsorptive-mediated endocytosis inhibitor), and MBCD (cholesterol-dependent membrane fusion inhibitor). As shown in Figure 3B, SLN-CMN preferred to cross the cell membrane through macropinocytosis, adsorptive- and caveolae-mediated endocytosis. Nonetheless, clathrin- and adsorptive-mediated endocytosis were the two major pathways of Lip-CMN (Figure 3C). Although the surface modification of SLN-CMN and Lip-CMN by the cationic peptides C, M, and N allowed these two nanomedicines to be internalized by adsorptive-mediated endocytosis into SAS cells, SLN-CMN and Lip-CMN apparently displayed different internalization mechanisms.

Endosomal escape is an important step for the successful delivery of nanoparticles to intracellular targets 37. The intracellular trafficking of internalized DNR/Lip-CMN and FAM-miR-200/SLN-CMN was observed by CLSM (Figures 3C-D). The DNR/Lip-CMN fluorescence (red) was co-localized with NG2 (stained with cyan-labeled anti-NG2 antibody) in SAS cells after 30 min of incubation. At 30 min, DNR/Lip-CMN was also co-localized with mitochondria (stained with MitoTracker as green; MitoGreen), which are typically located close to the nuclei. Particularly, DNR from Lip-CMN was predominantly co-localized with nuclei at 8 h, suggesting successful escape from the endosomes and/or lysosomes to its intracellular target (Figure 3D). For comparison, the trafficking of untargeted Lip and single-peptide-modified Lip was also investigated, and the results are shown in Figures S4A-D. We found that DNR/Lip-C enhanced Lip penetration into SAS cells to release DNR intracellularly (Figure S4B). DNR/Lip-M, with the mitochondrion-targeted peptide M, was verified to successfully transport DNR to mitochondria (Figure S4C) and thus activate the mitochondrion-associated apoptosis pathway (as shown below). The similar pro-apoptosis effect of M-peptide-linked nanostructures has also been reported to induce the mitochondrion-mediated apoptosis and intensify the anticancer efficacy of doxorubicin* in vitro* 30. Furthermore, the CLSM images confirmed that DNR/Lip-N was co-localized in SAS cells with NG2, a proteoglycan overexpressed in angiogenetic tumor cells (Figure S4D).

Additionally, FAM-miR-200/SLN-CMN (green) was co-localized mostly with NG2 of SAS cells at 10 min (Figure 3E). At 30 min, FAM-miR-200/SLN-CMN was distributed predominantly in the cytoplasm. Interestingly, FAM-miR-200 displayed an escalated extent of co-localization in mitochondria (stained with MitoRed) at 3 h (Figure 3E) at least partially because N peptide is a ligand that targets mitochondria. These results showed that SLN-CMN had good endosomal-escape capability and high accumulation in mitochondria and cytoplasm, thereby preventing miR-200 degradation in early endosomes and lysosomes.

### Toxicity to noncancerous and cancer cells

**Figure 4:**
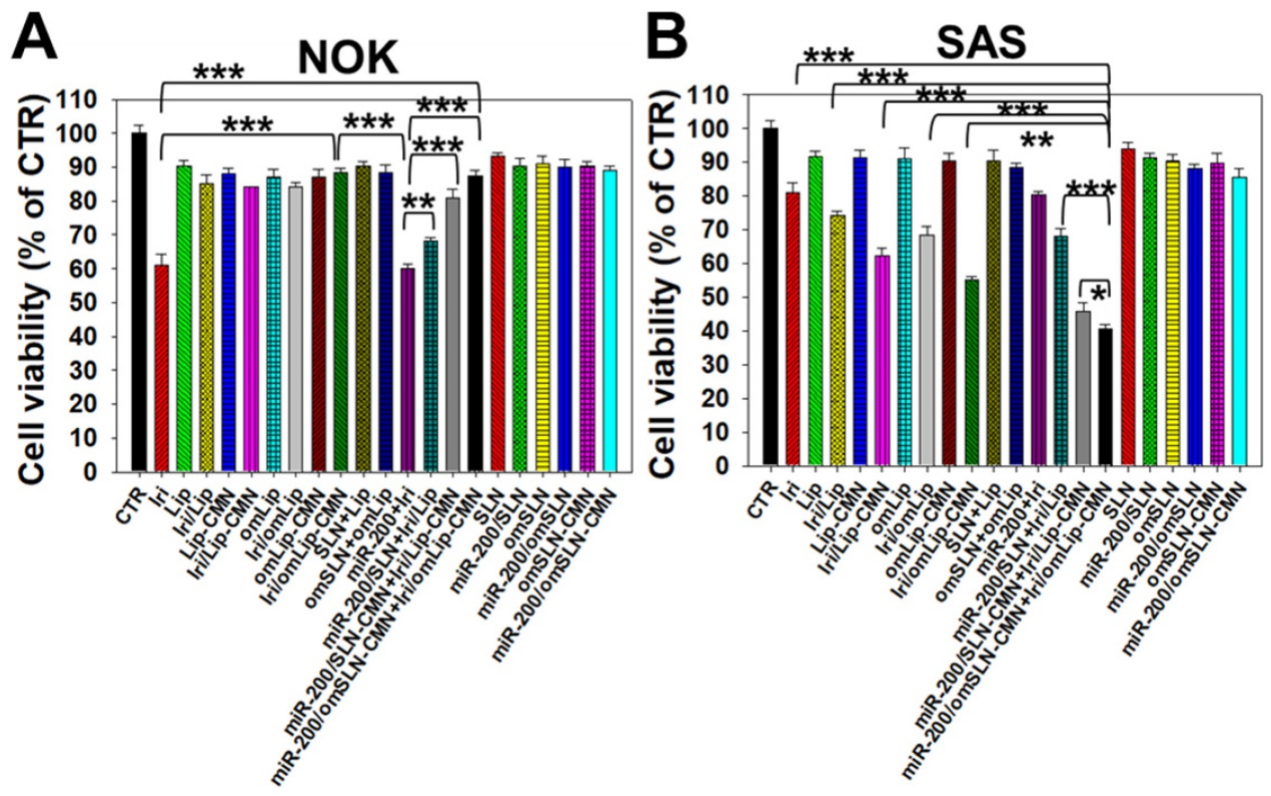
Cytotoxicity of various formulations on NOK and SAS cells. (A) NOK cells and (B) SAS cells were treated with different formulations (SAS = 0.7 μM; miR-200 = 100 nM) for 48 h, and cell viability was determined by SRB assay (*statistical significance at P < 0.05; ** P < 0.01; *** P < 0.001).

All SLN formulations in the presence or absence of miR-200 showed marginal toxicity to NOK cells (Figure 4A) as measured by SRB assay. The Iri solution displayed approximately 40% cytotoxicity to noncancerous NOK cells, but the toxicity of Iri/Lip, Iri/Lip-CMN, and Iri/omLip-CMN to NOK cells was lower than that of Iri solution (Figure 4A). The combined treatment of Iri/omLip-CMN and miR-200/omSLN-CMN did not increase the cytotoxicity to NOK cells (Figure 4A). This result suggested that these liposomal Iri formulations may diminish the possible Iri-induced side effects, such as oral mucositis, nausea, and vomiting 38. Iri and/or miR-200 in different formulations showed various cytotoxic effects on SAS cells, as observed by SRB assay (Figure 4B). We found that the initial administration of miR-200/SLN-CMN followed by Iri/Lip-CMN caused a significant reduction in cancer-cell viability (about 55% inhibition; Figures 4B and S5). The co-administration of miR-200 and Iri in one formulation such as SLN-CMN or Lip-CMN exhibited mild cytotoxicity on SAS cells (15%-20% inhibition on SAS cell viability; Figure S5). The simultaneous co-treatment of miR-200/SLN-CMN and Iri/Lip-CMN showed approximately 30% inhibition on SAS cells (Figure S5). Remarkably, the greatest growth inhibition of SAS cells (about 60% inhibition; Figure 4B) was found in the initial treatment with miR-200/omSLN-CMN followed by Iri/omLip-CMN formulation. These results indicated the necessity to pretreat SAS cells with miR-200/omSLN-CMN for 24 h to activate the critical anticancer signaling pathways (as verified below) and thus further enhance the cytotoxic effect of Iri/omLip-CMN on SAS cells (Figures 4B and S5).

### Enhanced apoptosis and related death mechanisms induced by the combined treatment in SAS cells

**Figure 5:**
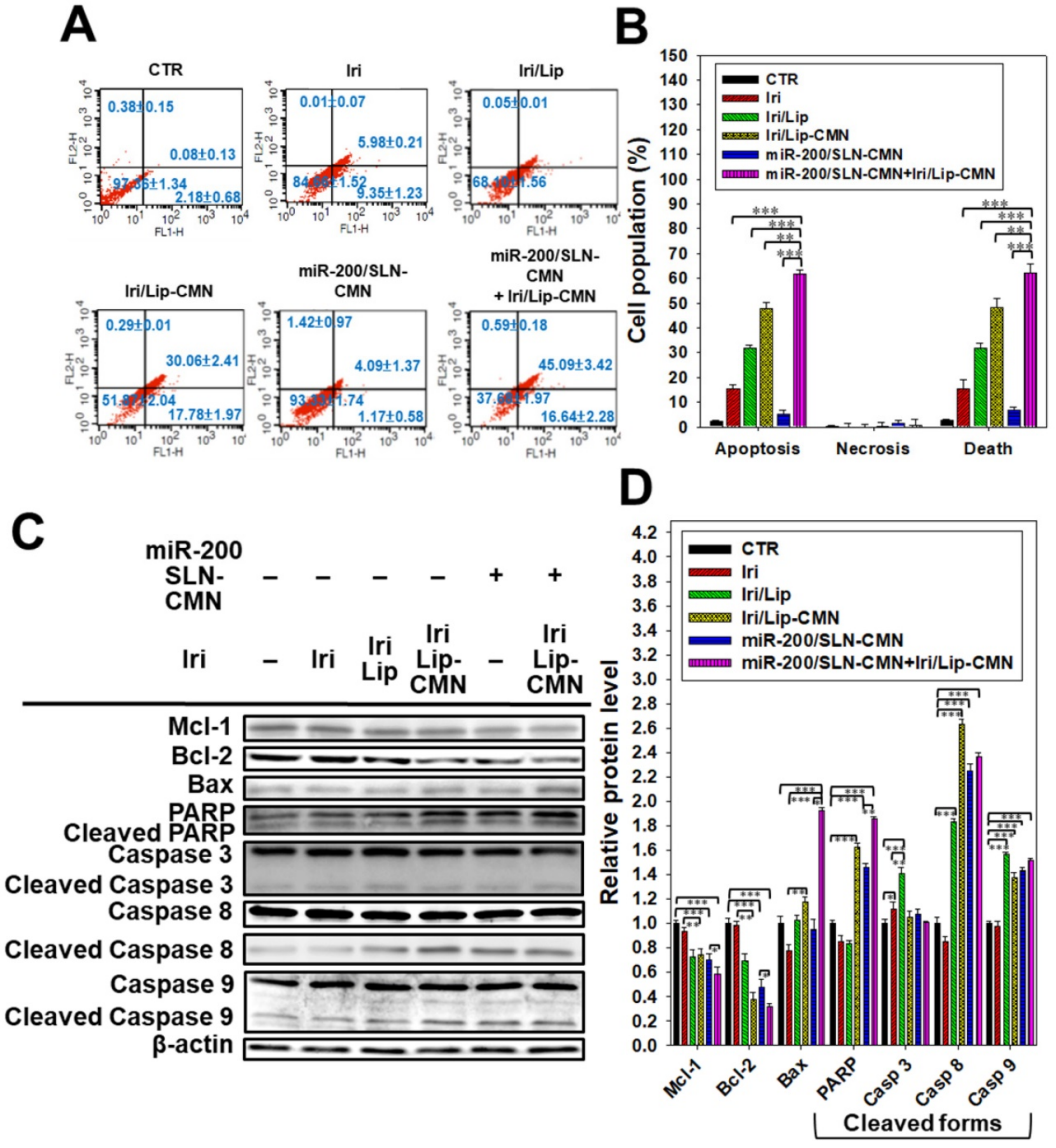
Anticancer effect of different formulations on the apoptosis pathway in SAS cells. (A) SAS cells were pretreated with miR-200/SLN-CMN (100 nM) for 24 h and then treated with irinotecan (Iri) or Iri/Lip-CMN (SAS = 0.7 μM) for 48 h. (B) Annexin V kit was used to detect and quantify the percentage of apoptotic cells (*statistical significance at P < 0.05; **P < 0.01; ***P < 0.001). (C-D) SAS cells were pretreated with miR-200/SLN-CMN (100 nM) for 24 h and then treated with Iri or Iri/Lip-CMN (SAS = 0.7 μM) for 48 h. Western blot assay was used to measure the protein levels on the apoptosis-associated pathway.

Iri causes cancer cell death by inhibiting Topo-I through the formation of stable Topo-I-DNA complexes 39. Its combined treatment with miR-200/SLN-CMN further exacerbated the apoptosis effects of Iri/Lip-CMN against SAS cells (Figures 5A-B). Our molecular-mechanism investigation by Western blotting indicated that after treatment with miR-200/SLN-CMN and Iri/Lip-CMN, the protein expression levels of Bax, cleaved PARP, and caspases 8 and 9 were remarkably upregulated, whereas the protein expression levels of Bcl-2 and Mcl-1 were considerably downregulated (Figures 5C-D). These results confirmed that Iri was an apoptosis inducer 9. The encapsulation in peptide-modified Lip (Iri/Lip-CMN) and the combined treatment with miR-200/SLN-CMN potentiated the ability of Iri to activate apoptosis. Lip-CMN or SLN-CMN may have enhanced the transport of Iri and/or miR 200 to mitochondria or the neighboring environment to activate the mitochondrion-related intrinsic apoptosis pathway and induce the extrinsic apoptosis pathway (Figure 5).

### miR-200/SLN-CMN as a migration inhibitor and chemotherapeutic sensitizer in SAS cells

**Figure 6:**
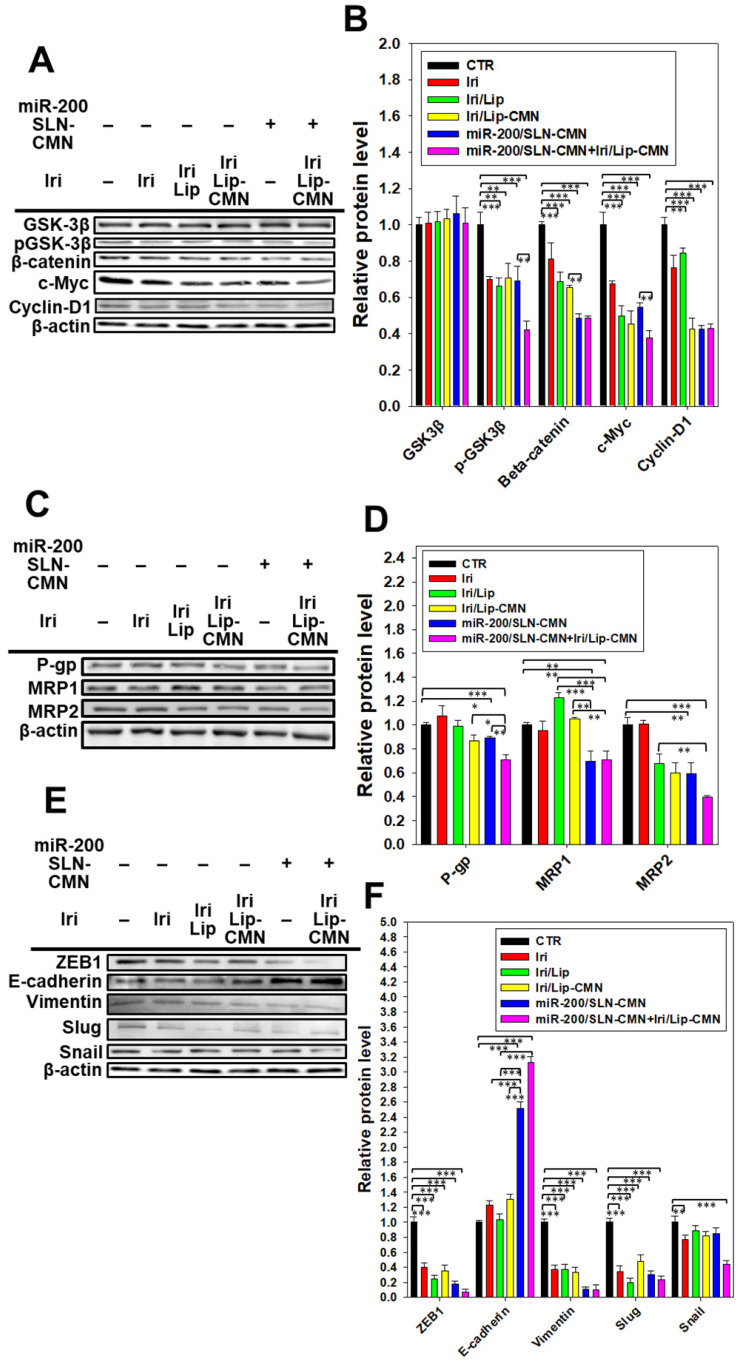

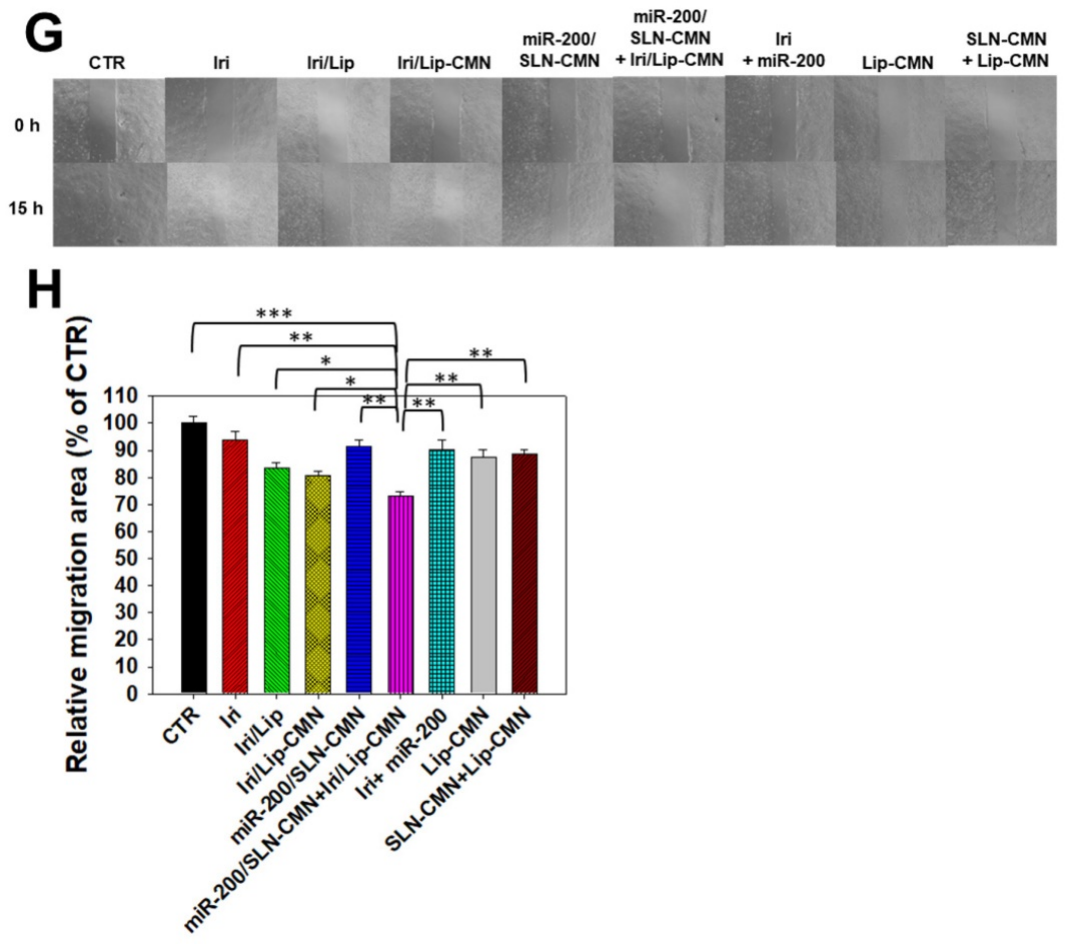
Effects of different formulations on diverse signaling pathways and relative migration percentage in SAS cells. SAS cells were pretreated with miR-200/SLN-CMN (100 nM) for 24 h and then with Iri or Iri/Lip-CMN (SAS = 0.7 μM) for 48 h. The protein expression levels of (A-B) Wnt/β-catenin, (C-D) MDR, and (E-F) EMT pathways were determined by Western blotting. (G) Cells were pretreated with different formulations for 15 h before the images were taken. (H) Quantification of the relative percentage of cell-migration area (*statistical significance at P < 0.05; **P < 0.01; ***P < 0.001).

miR-200 inhibits cancer-cell migration and invasion by targeting ZEB1 and 2 18. EMT, a crucial tumor-progression factor, promotes the exacerbation of metastasis, which is one of the major causes of cancer-associated death in patients 40. Furthermore, the Wnt/β-catenin signaling pathway is critical to the regulation of cell proliferation, differentiation, adhesion, resistance, and migration 41. The MDR pathway also plays an important role in drug resistance and treatment failure 42. MDR1 or ATP-binding cassette subfamily B member 1 (ABCB1; P-gp) and other MDR genes, such as ABCC1 (MRP1) and ABCC2 (MRP2), are at least partially regulated by the TCF4/β-catenin transcriptional complex and the associated β-catenin signaling pathway 43,44. The development of the EMT pathway required for metastasis is also related to the activated Wnt/β-catenin signaling pathway 45. For example, slug, snail, and vimentin are regulated not only by the EMT pathway but also by the β-catenin pathway 46. As shown in Figures 6A-B, the protein expression levels of p-GKS-3β, β-catenin, Cyclin D1, and c-Myc were markedly downregulated by miR-200/SLN-CMN. Furthermore, the combined treatment of miR-200/SLN-CMN and Iri/Lip-CMN demonstrated the highest inhibition (%) on these proteins. Interestingly, the protein expression levels of P-gp, MRP1, and MRP2 were also significantly inhibited when SAS cells were co-treated with miR-200/SLN-CMN and Iri/Lip-CMN (Figures 6C-D). As a result, miR-200 in SLN-CMN served as a chemotherapy sensitizer in SAS cells to increase the cytotoxicity of Iri loaded in Lip-CMN. Accordingly, the protein expression levels of E-cadherin (an epithelial marker) were enhanced, but those of various transcription factors such as ZEB1, slug, snail, and vimentin (mesenchymal markers) were considerably reduced by the combined treatment of miR-200/SLN-CMN and Iri/Lip-CMN (Figures 6E-F). The migration of cells treated with Iri/Lip-CMN was inhibited after 15 h of treatment (Figures 6G-H). Importantly, the combined treatment of miR-200/SLN-CMN and Iri/Lip-CMN further potentiated the extent of inhibition (Figures 6G-H). These findings suggested that the combined treatment of miR-200/SLN-CMN and Iri/Lip-CMN considerably repressed the EMT, MDR, and Wnt/β-catenin signaling pathways (Figures 6A-H). Collectively, our results indicated that miR-200 in SLN-CMN may modulate multiple pathways to suppress anti-apoptosis, proliferation, chemoresistance, and EMT in SAS cells. Consistently, miR-200 upregulation has been demonstrated to inhibit cyclin D1, Bcl‑2, and N‑cadherin, as well as the transcription factors Snail, Slug, and ZEB1, in different cancer types 47,48.

### *In vivo* antitumor efficacy and IVIS imaging studies involving SAS tumor-bearing mice

**Figure 7:**
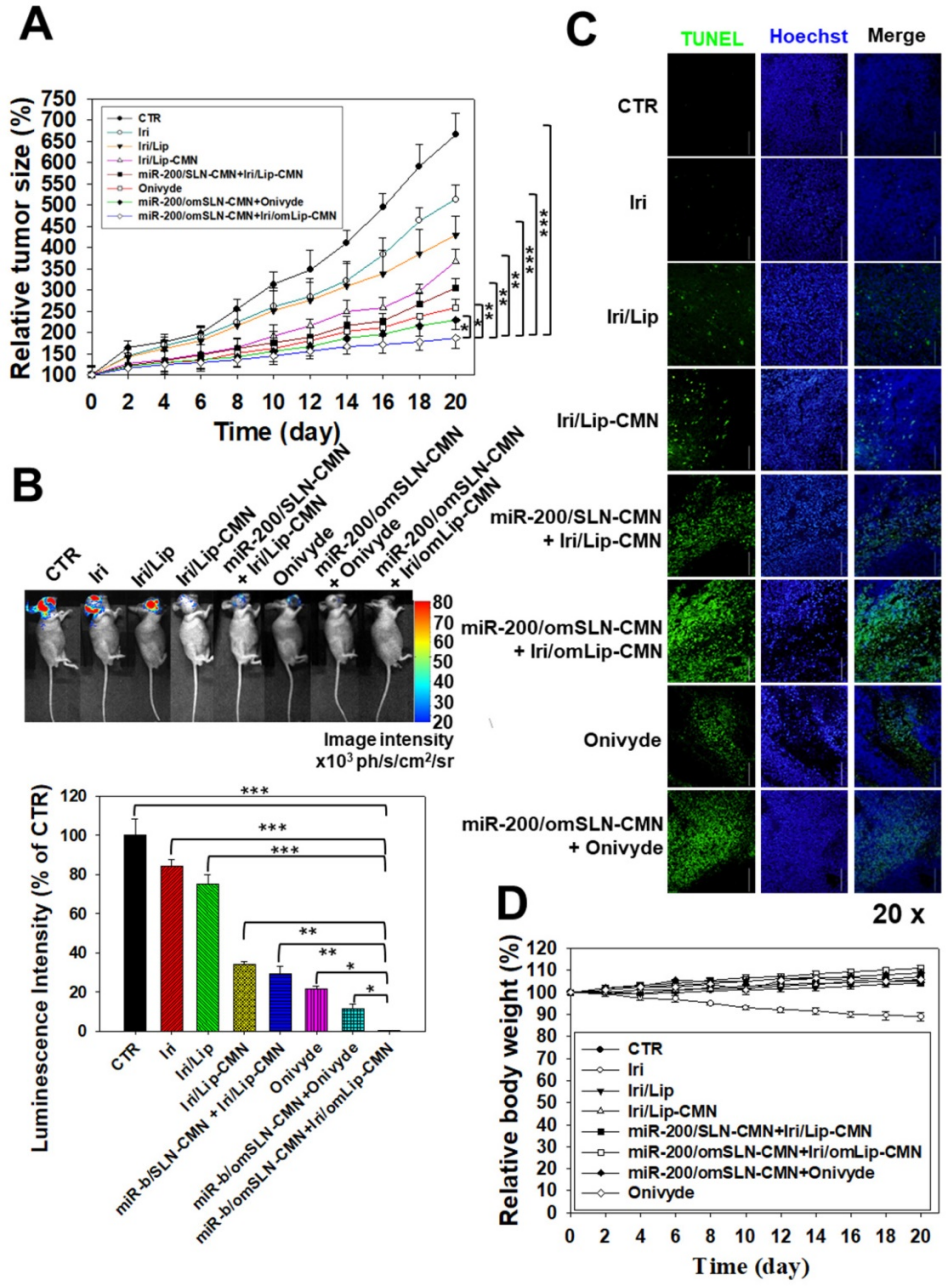
Antitumor efficacy of different formulations and relative body weight percentage on SAS/luc-bearing mice. (A) Antitumor efficacy of SAS/luc-bearing mice intravenously injected with different formulations. Tumor growth was measured with digital calipers every 2 days (*statistical significance at P < 0.05; **P < 0.01; ***P < 0.001). (B) IVIS images of SAS/*luc*-bearing mice treated with different formulations. (C) TUNEL analysis of *in vivo* apoptosis in SAS tumor cells (green) on the day after the last administration. Nuclei (blue) were stained with Hoechst. Scale bar, 100 μm. (D) Body weight of SAS/*luc*-bearing mice treated with different formulations for 20 days.

A SAS/luc tumor-bearing mouse model was established to determine the antitumor efficacy of different formulations *in vivo*. The combined treatment of pH-sensitive miR-200/omSLN-CMN + Iri/omLip-CMN displayed the most significant inhibitory effect on SAS-bearing mice, revealing the superior antitumor efficacy of this combined nanoparticle formulation among the various treatment groups (Figure 7A). Furthermore, the mice bearing the SAS tumor without treatment (CTR) displayed high bioluminescence intensity in the tumor region as shown by the IVIS images (Figure 7B). For comparison, the images were recorded with IVIS software, and the relative bioluminescence intensity is shown in the lower panel of Figure 7B. The bioluminescence intensity of the SAS tumor-bearing mice treated with various Iri and miR-200 formulations decreased, indicating the different tumor sizes (Figure 7B). Mice treated with miR-200/omSLN-CMN + Iri/omLip-CMN demonstrated the most substantial decrease in tumor size and luminescence intensity (Figures 7A-B). Interestingly, the antitumor efficacy of miR-200/omSLN-CMN + Iri/omLip-CMN was superior even to that of miR-200/omSLN-CMN + Onivyde. This finding was consistent with the TUNEL assay results (Figure 7C), which indicated that tumor-cell apoptosis and necrosis (green) were greatly intensified in the miR-200/omSLN-CMN + Iri/omLip-CMN group compared with those in the other groups. By contrast, *in vivo* TUNEL assay results (Figures S6A-B) verified that the apoptosis and necrosis of vessel cells (green) were most remarkably reduced in the miR-200/omSLN-CMN + Iri/omLip-CMN group. This finding suggested that miR-200/omSLN-CMN + Iri/omLip-CMN co-treatment had the lowest toxicity to vessel cells among all treatment groups. Notably, its toxicity was lower than that of Onivyde or miR-200/omSLN-CMN + Onivyde (Figure S6).

The evaluation of various formulations on body weight indicated that SAS-bearing mice treated with Iri showed a constant decrease in body weight (Figure 7D). All other groups exhibited mild escalations in body weight with slight individual differences (Figure 7D). Nevertheless, the curve of survival percentage demonstrated that Iri without nanoformulation caused 20% death of tumor-bearing mice (Figure S7) possibly because of the Iri-associated toxicity and the continuous body-weight loss during the 20 days. All treatment groups with nanocarriers maintained 100% survival throughout the 20-day period (Figure S7), suggesting the safe compatibility of these delivery systems with the clinically available Onivyde.

### *In vivo* biosafety evaluation: biochemical tests and H&E staining

**Figure 8:**
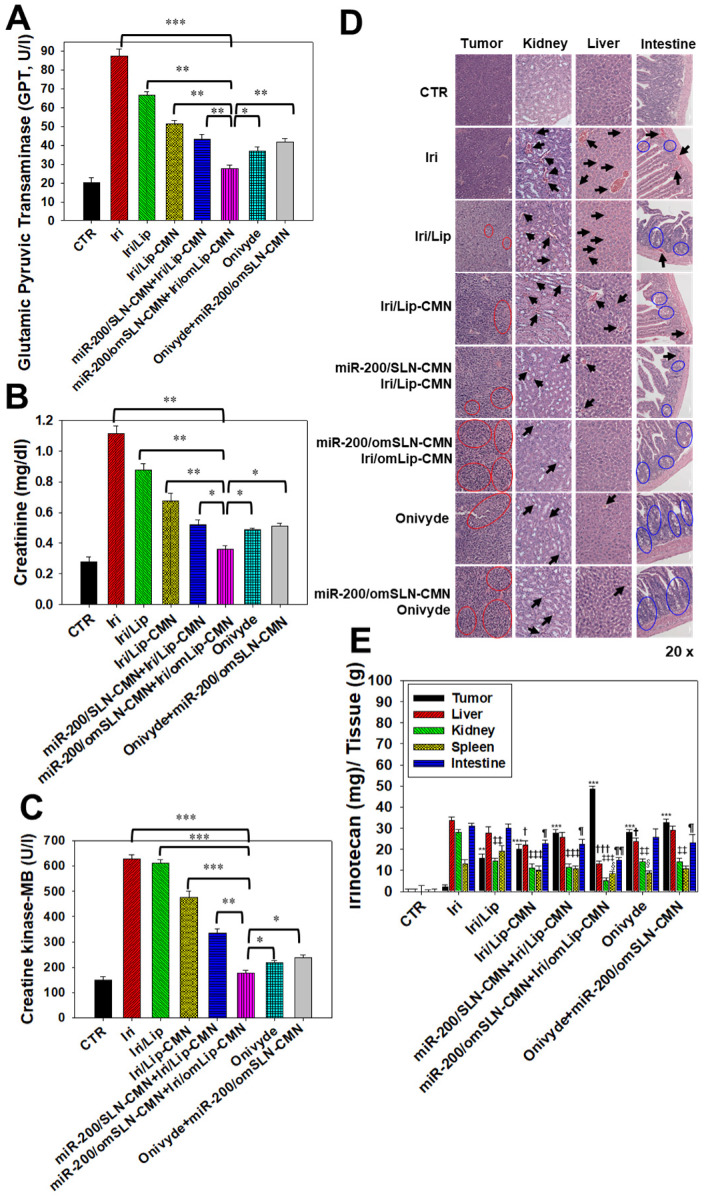

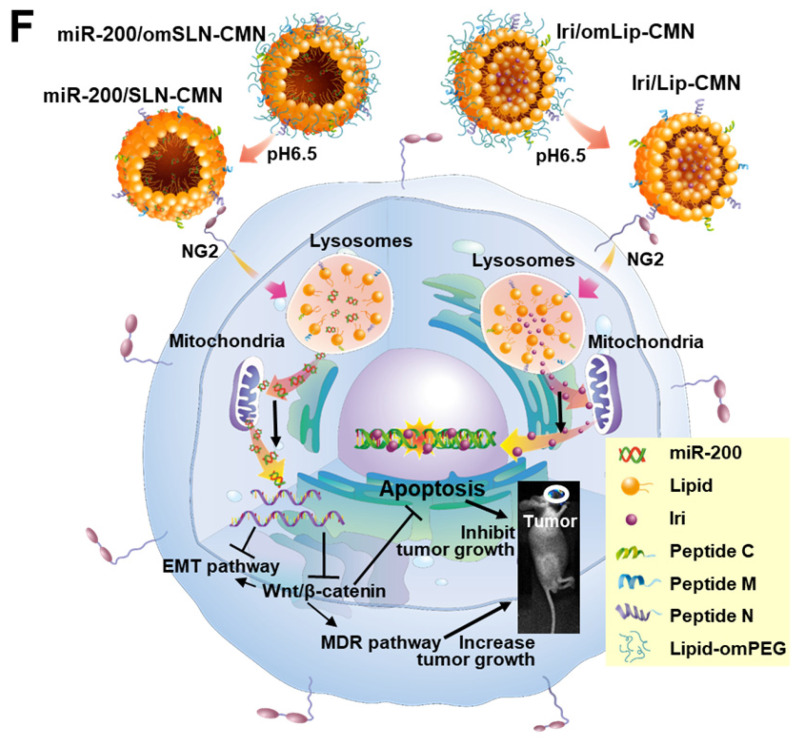
Biosafety and biodistribution studies on different formulations of SAS-bearing mice. (A-C) Blood biochemical indices of the (A) liver, (B) kidney, and (C) heart of mice treated with different formulations. *Statistical significance at P < 0.05; **P < 0.01; ***P < 0.001. (D) Histological photomicrographs of the tumor, kidney, liver, and intestinal sections stained with H&E. Red circles indicate necrosis or apoptosis; black arrows denote inflammation; blue circles correspond to cell injury. (E) Biodistribution study of different irinotecan formulations in SAS-bearing mice by using a UV spectrophotometer. ^*^P < 0.05; ^**^P < 0.01; ^***^P < 0.001 compared with Iri group for tumor.^ †^P < 0.05; ^††^P < 0.01; ^†††^P < 0.001 compared with Iri group for liver.^ ‡^P < 0.05; ^‡‡^P < 0.01; ^‡‡‡^P < 0.001 compared with Iri group for kidney.^ §^P < 0.05; ^§§^P < 0.01; ^§§§^P < 0.001 compared with Iri group for spleen. ^¶^P < 0.05; ^¶¶^P < 0.01; ^¶¶¶^P < 0.001 compared with Iri group for intestine. (F) Schematic of the molecular mechanisms by which miR-200/omSLN-CMN and Iri/omLip-CMN regulated multiple signaling pathways in the HNC model. NG2: nerve/glial antigen 2.

Biochemical tests and H&E staining were conducted to detect organ functions and the histopathological characteristics of the various formulations used (Figures 8A-D). Serum GPT, CRE, and CK-MB, which are important biomarkers of the liver, kidney, and heart functions, respectively, were examined 1 day after the final treatment. Results showed that their levels considerably increased after the Iri treatments (Figures 8A-C), indicating the substantial damage inflicted by Iri to the liver, kidney, and heart. This finding may also partially explain the 20% death in the Iri-treated group of tumor-bearing mice (Figures S7). However, the serum levels of GPT, CRE, and CK-MB increased less after treatment with Iri in various liposomal formulations compared with the Iri group. In particular, these three biochemical markers maintained levels similar to those of the CTR groups (P > 0.05) after co-treatment with miR-200/omSLN-CMN + Iri/omLip-CMN (Figures 8A-C). The combined treatment of miR-200/omSLN-CMN and Iri/omLip-CMN displayed the lowest toxicity to the liver, kidney, and heart among all treatment groups, and the degree of toxicity was even lower than that of Onivyde alone or Onivyde + miR-200/omSLN-CMN (Figures 8A-C). This result was partially due to the design of the pH-sensitive omPEG layer in omSLN-CMN and omLip-CMN to prolong circulation time, promote pH-sensitive tumor accumulation, and induce the exposure of targeting peptides for the improved uptake and release of miR-200 and Iri into intracellular target sites of the cytoplasm and nucleus. These beneficial effects rendered the combined treatment more effective than Onivyde and reduced the Iri-inflicted damages to vital organs or tissues.

Additionally, results of *in vivo* HE staining of tumor tissues indicated that the CTR group had more tumor cells and larger nuclei than the other treatment groups (Figure 8D; panel 1). However, the tumor tissues of the miR-200/omSLN-CMN + Iri/omLip-CMN group showed the most obvious phenomenon of pyknosis, i.e., nuclear chromatin condensation (red circles; Figure 8D), which indicated tumor apoptosis and necrosis (Figure 8D; panel 1). The potential toxicity of these Iri and/or miR-200 formulations on the kidney, liver, and intestines was further examined through H&E staining (Figure 8D; panels 2-4). For comparison, the H&E staining of the kidney, liver, and intestines of the CTR groups exhibited integral cell morphology (Figure 8D, panels 2-4). The intestinal, renal, and liver tissues of all treatment groups demonstrated interstitial hemorrhage (arrows), representing different degrees of tissue inflammation. The intestinal tissues also showed cell swelling, numerous vacuoles (potential indication of fatty degeneration; blue circles), and disordered cell arrangement, suggesting possible intestinal injury and inflammation (Figure 8D, panel 4). Interestingly, our *in vivo* results also indicated that miR-200/omSLN-CMN + Iri/omLip-CMN damaged the intestinal, renal, or liver cells to a lower extent and induced only minor histopathological abnormalities compared with the other treatment groups (Figure 8D, panels 2-4). Moreover, treatment with miR-200/omSLN-CMN + Iri/omLip-CMN confirmed a notable lessening of interstitial hemorrhage and tissue degeneration, which were most serious in the Iri group. Thus, tissue injury and inflammation were considerably more alleviated by the co-treatment of Iri/omLip-CMN and miR-200/omSLN-CMN than by Iri alone (Figure 8D, panels 2-4).

The biodistribution of Iri of different formulations in SAS-bearing mice was detected, and results demonstrated that Iri solution was distributed mainly in the liver, kidney, and intestines (Figure 8E). Nevertheless, miR-200/SLN-CMN + Iri/Lip-CMN, miR-200/omSLN-CMN + Iri/omLip-CMN, and Onivyde were primarily distributed in tumor tissues, particularly miR-200/SLN-CMN + Iri/Lip-CMN decorated with the pH-sensitive om-PEG (Figure 8E).

Considering the unmet medical need for HNC treatment, effective therapies should be based on the genetic or pathological characteristics or patient treatment history of various HNC types. SAS is a poorly differentiated, tumor-progression model of OSCC with aggressive invasion and migration 4, 49. Thus, the encouraging preclinical findings in the current study suggested that the excellent tumor-accumulating feature of miR-200/SLN-CMN + Iri/Lip-CMN with its tumor-detachable PEG-lipid derivative layer may specifically deliver miR-200 and Iri into HNC tumor sites to modulate multiple signaling pathways and efficiently suppress tumor migration, resistance, and metastasis. Future work must focus on overcoming challenges such as scaling up/manufacturing and multifarious clinical trial requirements to enable the extensive clinical use of these two nanoparticle formulations. The schematic of the molecular mechanisms by which miR-200/omSLN-CMN and Iri/omLip-CMN regulated multiple signaling pathways in the HNC model is shown in Figure 8F.

## Conclusion

A novel combination therapy was developed on the basis of the delivery of miR-200 and Iri by the corresponding carrier systems for the potential treatment of HNC. The functions of these nanoparticles were as follows: (1) decreasing noncancerous cellular uptake through the protection provided by the outer cleavable PEG-lipid shell, (2) enhancing passive tumor targeting via the EPR effect, and (3) improving active tumor targeting via specific ligand-receptor binding. The apoptosis of HNC cells treated with the combinatorial therapy was significantly induced by regulating various pathways, such as the Wnt/β-catenin, MDR, and EMT pathways. The therapeutic efficacy and safety of co-treatment with pH-sensitive and peptide-conjugated nanoparticles were better than those of the commercially available Onivyde and other formulations in tumor-bearing HNC mice. Overall, this study suggested that chemo- and gene therapy co-treatment with pH-sensitive and targeting peptide-modified nanoparticles may be an innovative strategy for HNC treatment.

## Supplementary Material

Supplementary figures and tables.

## Figures and Tables

**Table 1:** Characterization of various Lip and SLN formulations

Formulations	Particle size (nm)	PDI	Zeta potential (mV)	Encapsulation; efficiency (%)	Drug-loading; capacity (%)
Lip	164.8 ± 3.41	0.17 ± 0.02	-9.17 ± 1.68	—	—
Lip-CMN	166.3 ± 3.11	0.13 ± 0.05	-8.21 ± 1.19	—	—
omLip-CMN	170.2 ± 1.26	0.15 ± 0.04	-9.88 ± 1.42	—	—
Iri/Lip-CMN	172.1 ± 2.34	0.17 ± 0.05	-7.23 ± 1.26	85.89 ± 2.18	18.79 ± 1.22
Iri/omLip-CMN	175.2 ± 2.27	0.16 ± 0.04	-8.18 ± 1.43	86.28 ± 2.29	19.88 ± 1.03
SLN	133.8 ± 1.65	0.16 ± 0.07	20.47 ± 2.21	—	—
SLN-CMN	136.7 ± 2.25	0.21 ± 0.05	22.15 ± 1.25	—	—
omSLN-CMN	139.9 ± 2.76	0.18 ± 0.04	18.20 ± 1.49	—	—
miR-200/SLN-CMN	143.1 ± 1.18	0.19 ± 0.04	17.88 ± 1.38	86.78 ± 0.56	18.34 ± 1.23
miR-200/omSLN-CMN	148.6 ± 0.36	0.12 ± 0.05	16.70 ± 1.69	87.19 ± 0.81	19.52 ± 1.56

## Abbreviations

HNC: head and neck cancer
SAS: human tongue squamous carcinoma cell line
miR: microRNA
Iri: irinotecan
Topo: topoisomerase
MDR: multidrug resistance
P-gp: P-glycoprotein
MRP: MDR-associated proteins
EMT: epithelial-mesenchymal transition
ZEB1: zinc finger E-box binding homeobox 1
SLN: solid lipid nanoparticles
Lip: liposomes
CPP: cell-penetrating peptide
NG2: nerve/glial antigen 2
N peptide: a NG2-binding peptide
M peptide: one mitochondrion-directed apoptosis-inducing peptide
C peptide: one cell-penetrating peptide with high potency and selectivity against cancer cells
omPEG: O′-methyl polyethylene glycol
omSLN-CMN and omLip-CMN: SLN and Lip modified with pH-sensitive PEG-lipid derivative (lipid-imine-omPEG) and peptide C, M, and N
EPR: enhanced permeability and retention
MALDI-TOF MS: matrix-assisted laser desorption ionization time-of-flight mass spectrometry
TEM: transmission electron microscopy
EE%: encapsulation efficiency
DL%: drug-loading capacity
PDI: polydispersity index
NOK: normal oral keratinocyte
FBS: fetal bovine serum
DMA: 5-(N,N-dimethyl) amiloride
CPZ: chlorpromazine
CLSM: confocal laser scanning microscopy
SRB: sulforhodamine B
PI: propidium iodide
SDS-PAGE: sodium dodecyl sulfate-polyacrylamide gel
CTR: control
H&E: hematoxylin and eosin
GPT: glutamate pyruvate transaminase
CRE: creatinine
CK-MB: creatine kinase-MB
DNR: daunorubicin
LysoGreen: LysoTracker® Green
TUNEL: terminal deoxynucleotidyl transferase dUTP nick end labeling
ABC: ATP-binding cassette
