# Supplementary material for: PEG-coated nanoparticles detachable in acidic microenvironments for the tumor-directed delivery of chemo- and gene therapies for head and neck cancer
Source: Theranostics. 2020 May 17;10(15):6695–714. doi: 10.7150/thno.45164 (PMC7295054; doi:10.7150/thno.45164)
Supplement: Supplementary file 1 — Supplementary figures and tables. [file thnov10p6695s1.pdf]

Supporting Information

PEG-coated nanoparticles detachable in acidic microenvironments for the tumor-directed delivery of chemo- and gene therapies for head and neck cancer

Yu-Li Lo[1,2,3,*], Chih-Hsien Chang[1], Chen-Shen Wang[1], Muh-Hwa Yang[4,5], Anya Maan-Yuh Lin[1,2,6], Ci-Jheng Hong[1], Wei-Hsuan Tseng[1]

[1]Institute of Pharmacology, National Yang-Ming University, Taipei 11221, Taiwan

[2]Faculty of Pharmacy, National Yang-Ming University, Taipei 11221, Taiwan

[3]Center for Advanced Pharmaceutics and Drug Delivery Research, National Yang-Ming University, Taipei 11221, Taiwan

[4]Institute of Clinical Medicine, National Yang-Ming University, Taipei 11221, Taiwan

[5]Division of Medical Oncology, Department of Oncology, Taipei Veterans General Hospital, Taipei 11217, Taiwan

[6]Department of Medical Research, Taipei Veterans General Hospital, Taipei 11217, Taiwan

*Corresponding author: E-mail address: yulilo@ym.edu.tw (Y.-L. Lo)

Supplementary Figures

(A)

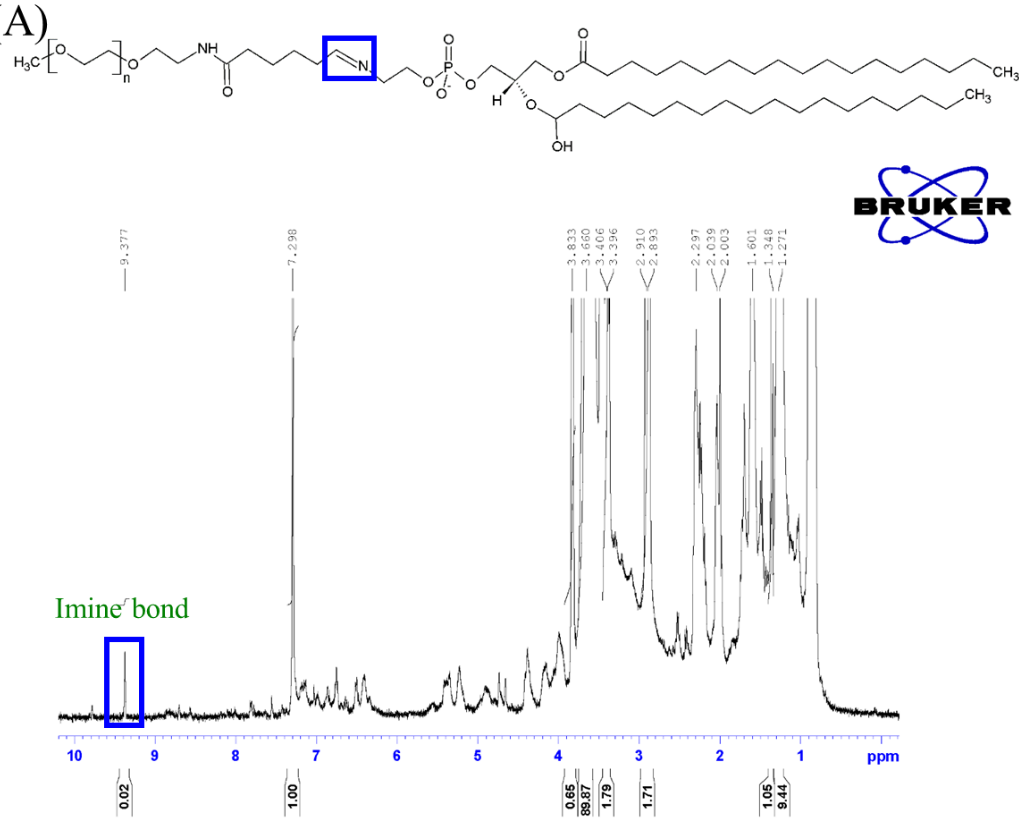

(B)

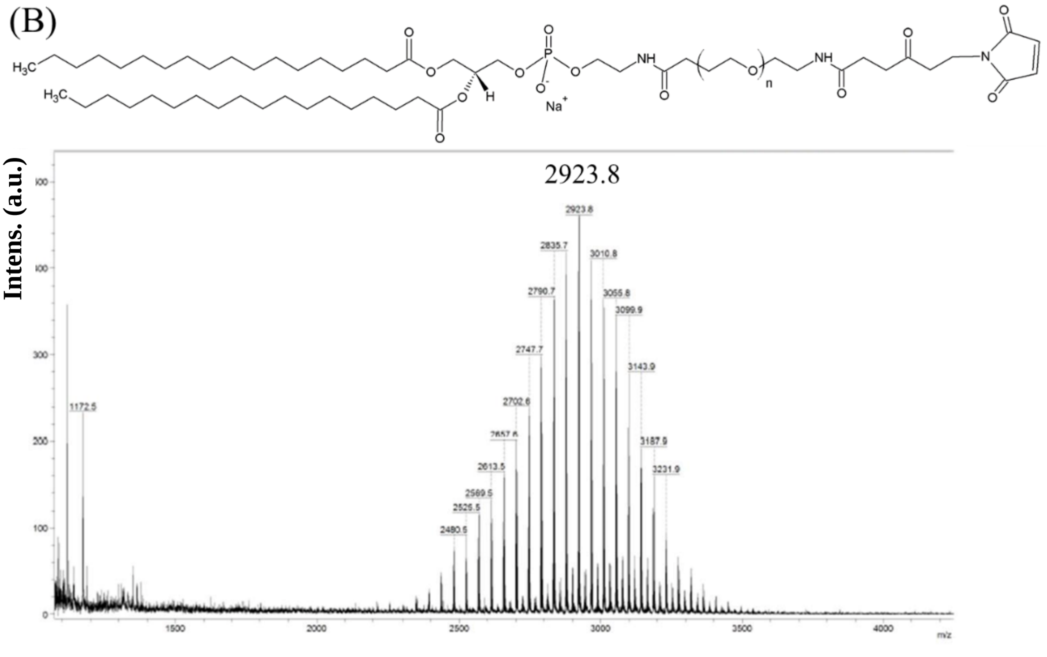

(C)

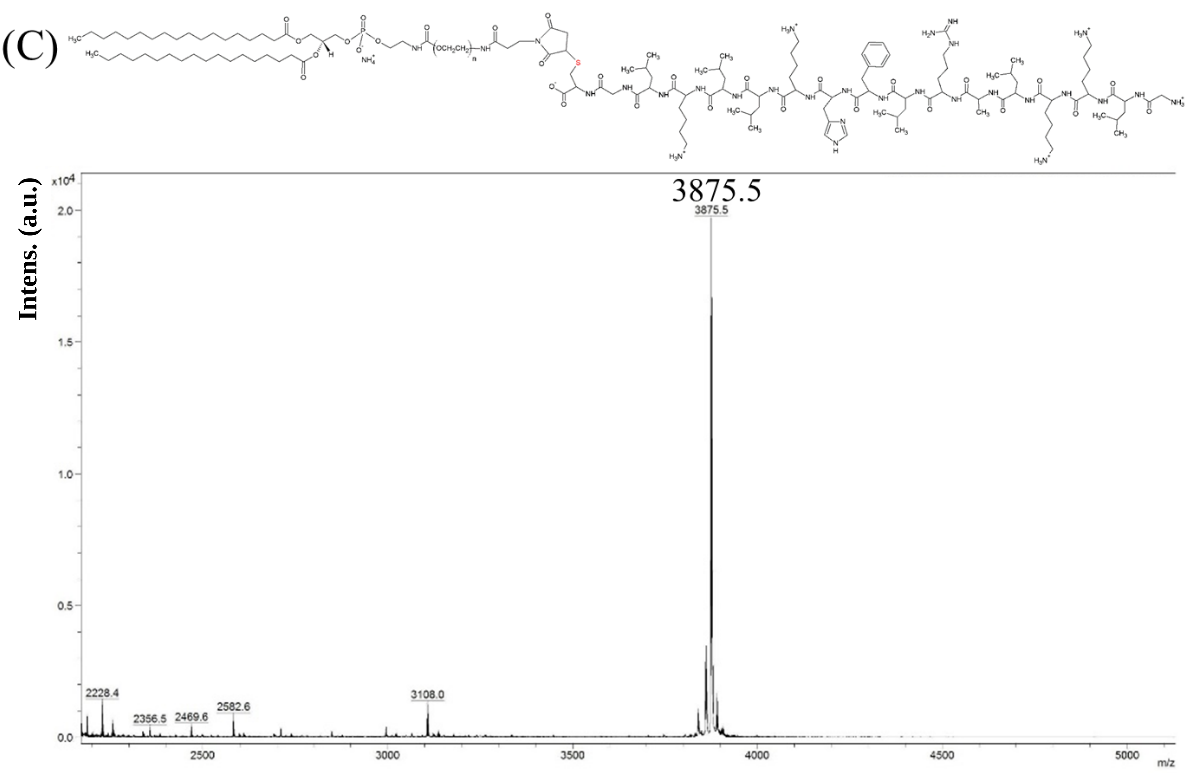
3875.5
Intens. (a.u.)
x10^4
2.0
1.5
1.0
0.5
0.0
3875.5
2228.4
2356.5
2469.6
2582.6
3108.0
2500
3000
3500
4000
4500
5000
m/z

(D)

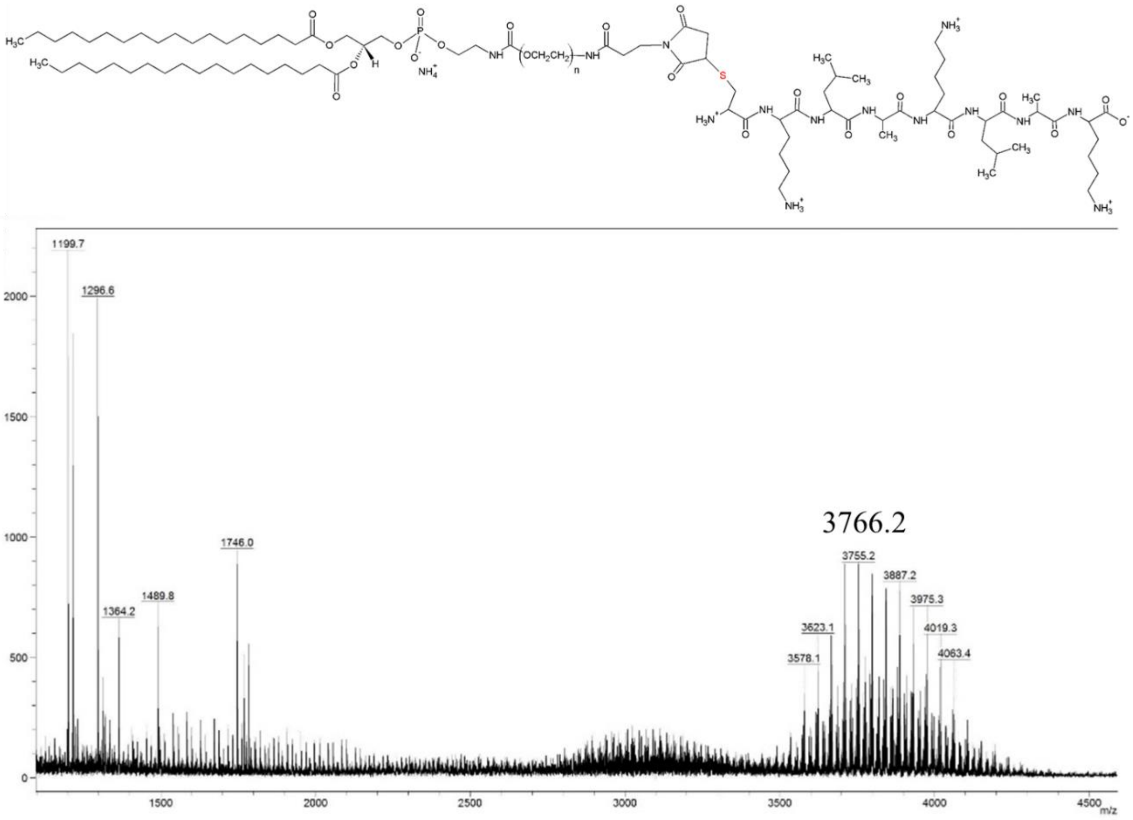
Intens. (a.u.)
2000
1500
1000
500
0
1199.7
1296.6
1364.2
1489.8
1746.0
3766.2
3755.2
3887.2
3975.3
4019.3
4063.4
3623.1
3578.1
1500
2000
2500
3000
3500
4000
4500
m/z

(E)

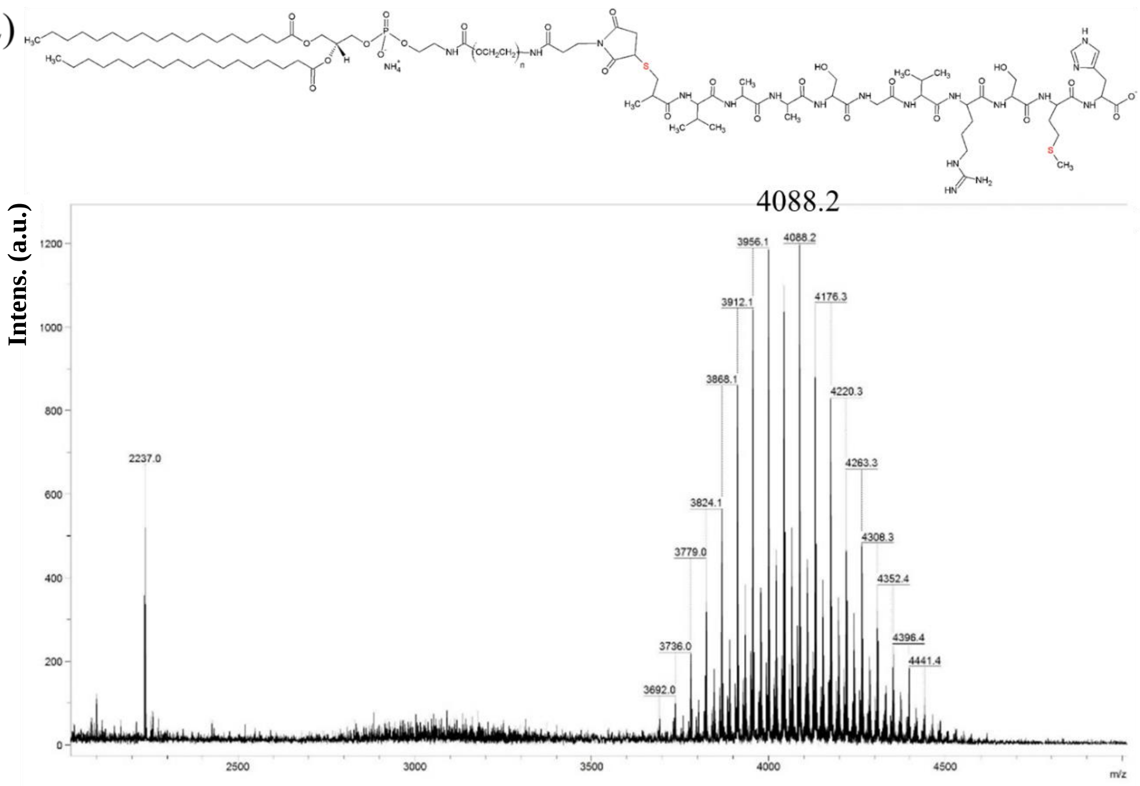

Figure S1. NMR and mass spectrometric characterization of DSPE-omPEG and peptide-modified DSPE-PEG. (A) DSPE was conjugated to omPEG in organic solvent for 24h and the product DSPE-omPEG was detected by ^{1}H NMR. (B-E) Different peptides were conjugated to DSPE-PEG-maleimide. The mass spectra of (B) DSPE-PEG-maleimide and the products of (C) DSPE-PEG-N, (D) DSPE-PEG-M, and (E) DSPE-PEG-C were detected by MALDI-TOF.

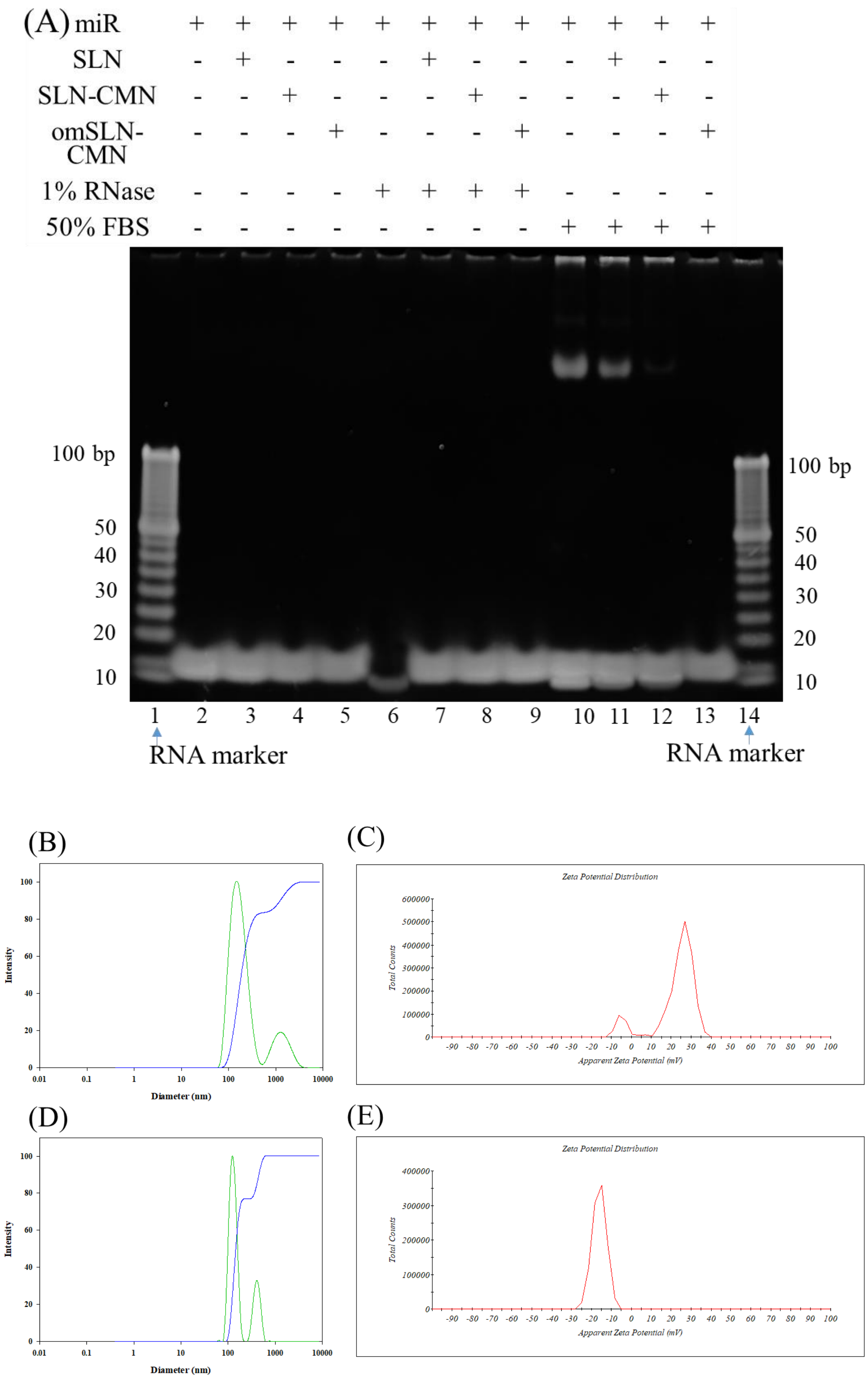

Figure S2. Protection test of miR-loaded formulations by gel retardation assay and characterization of miR-200/omSLN-CMN and Iri/omLip-CMN at pH 6.5. (A) miR was encapsulated with or without different SLN formulations (SLN, SLN-CMN, and omSLN-CMN) and incubated with 1%

RNase or 50% FBS at 37°C for 24 h. The samples were loaded into the gel and run by 8% polyacrylamide at 60 V. After staining with 0.001% ethidium bromide for 30 min at 25°C, the gel was then monitored and scanned using a gel documentation system (DigiGel; TopBio, Taipei, Taiwan). (B-E) Sizes and zeta potential of (B-C) miR/omSLN-CMN and (D-E) Iri/omLip-CMN were measured using a Zetasizer at pH 6.5.

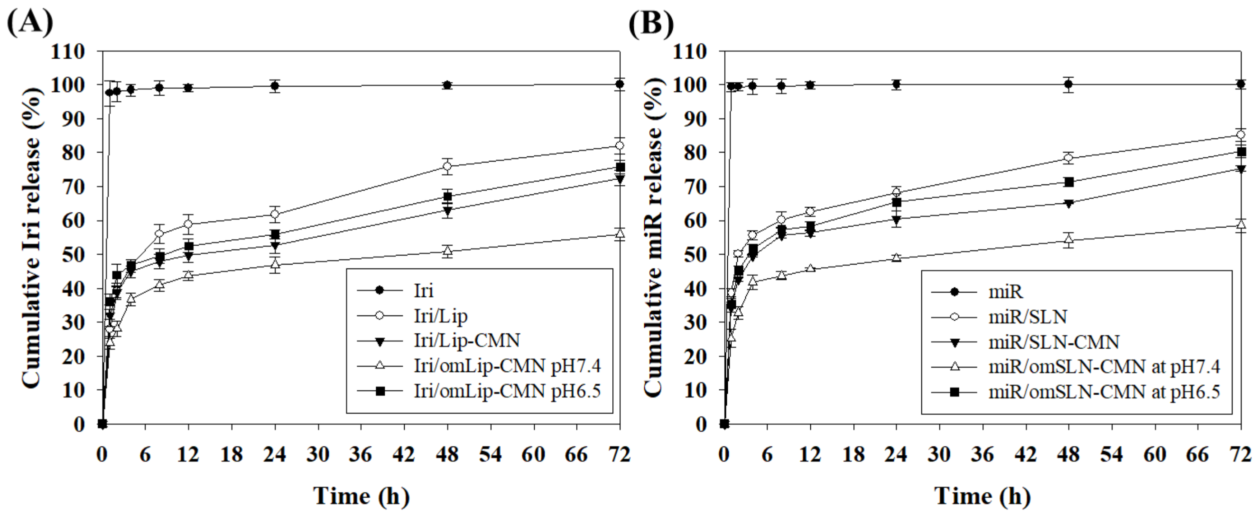

Figure S3. Release profiles of various formulations of (A) irinotecan and (B) miR at 37˚C.

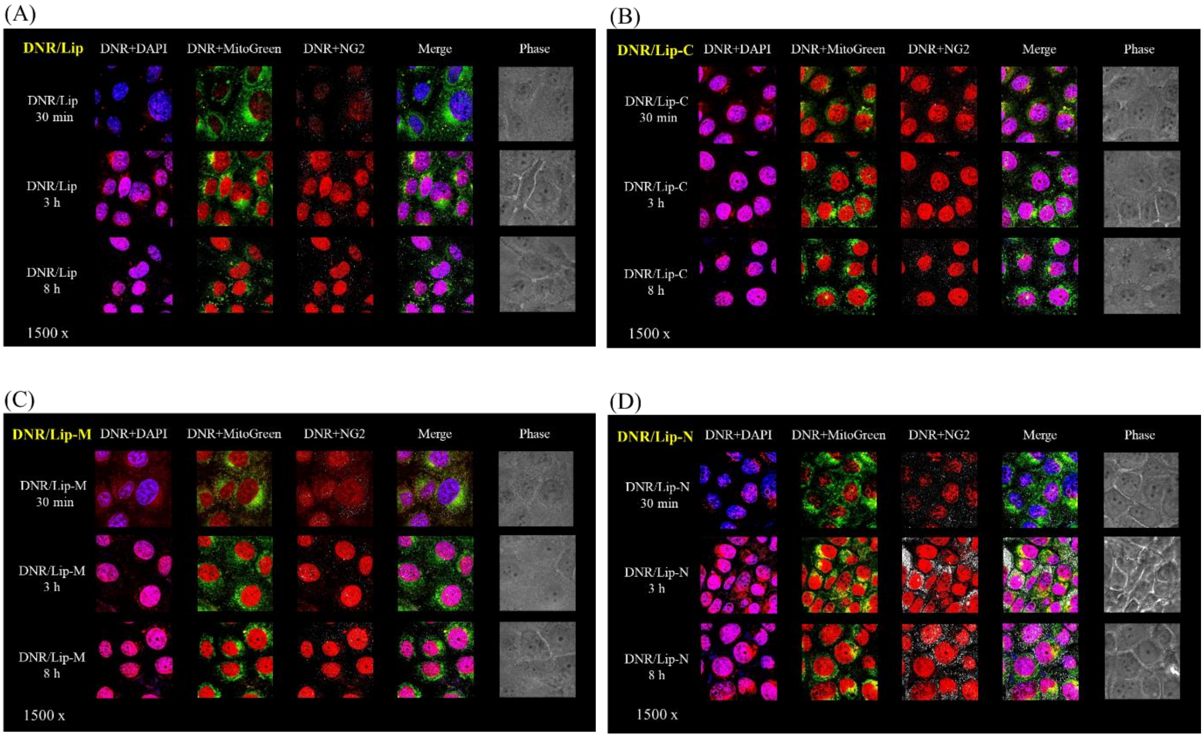

Fig. S4. (A) DNR/Lip, (B) DNR/Lip-C, (C) DNR/Lip-M, and (D) DNR/Lip-N were added to the cells for 30 min, 3 h, and 8 h. Intracellular localization of individual DNR/Lip formulations in SAS cells was observed by CLSM. Blue: DAPI (a nuclear dye); Green: MitoGreen (MitoTracker Green; a mitochondrial dye); Red: DNR; Gray: NG2 (nerve/glial antigen 2).

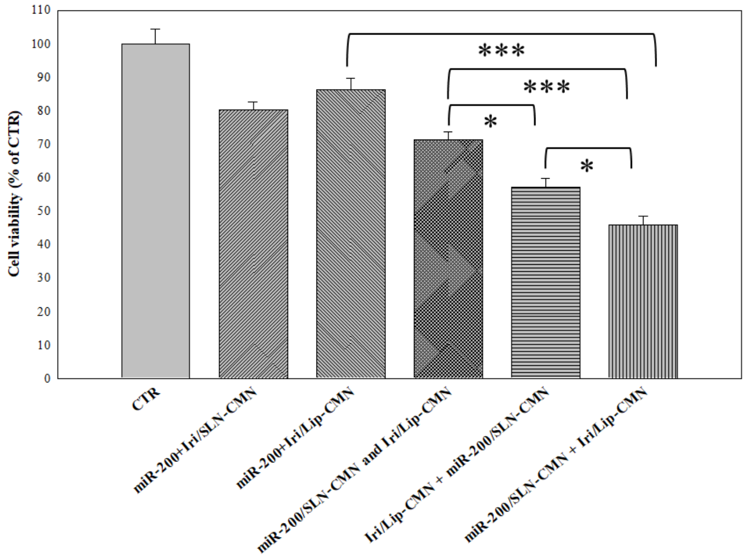

Figure S5. Evaluation of treatment order on cytotoxicity of different formulations in SAS cells for 48h by sulforhodamine B assay. *P < 0.05; **P < 0.01; ***P < 0.001

miR-200 + Iri/SLN-CMN: miR-200+ Iri together in SLN-CMN for 48 h;

miR-200 + Iri/Lip-CMN: miR-200+ Iri together in Lip-CMN for 48 h;

miR-200/SLN-CMN and Iri/Lip-CMN: miR-200/SLN-CMN and Iri/Lip-CMN together for 48 h;

Iri/Lip-CMN + miR-200/SLN-CMN: Iri/Lip-CMN for 24 h and followed by miR-200/SLN-CMN for 24 h;

miR-200/SLN-CMN + Iri/Lip-CMN: miR-200/SLN-CMN for 24 h and followed by Iri/Lip-CMN for 24 h.

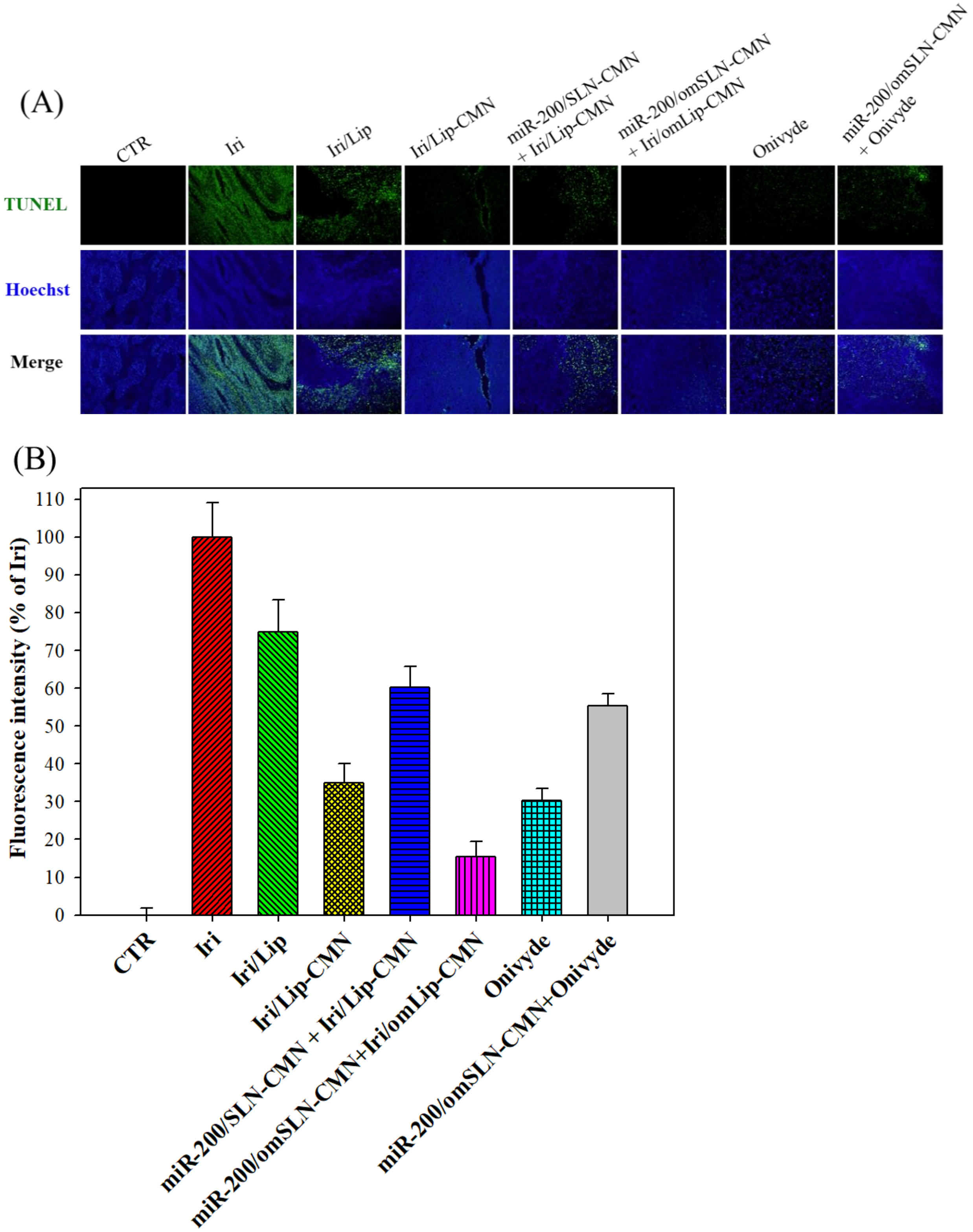

Figure S6. In vivo apoptosis evaluation in vessel cells of SAS/luc-bearing mice. (A) TUNEL analyses of *in vivo* apoptosis evaluation by different formulations on vessel cells of SAS/luc-bearing mice on the day after the last administration. Nuclei were stained with Hoechst (blue). Scale bar, 100 μm. (B) Relative apoptosis % of vessels cells was measured by the relative green fluorescence intensity of TUNEL assay.

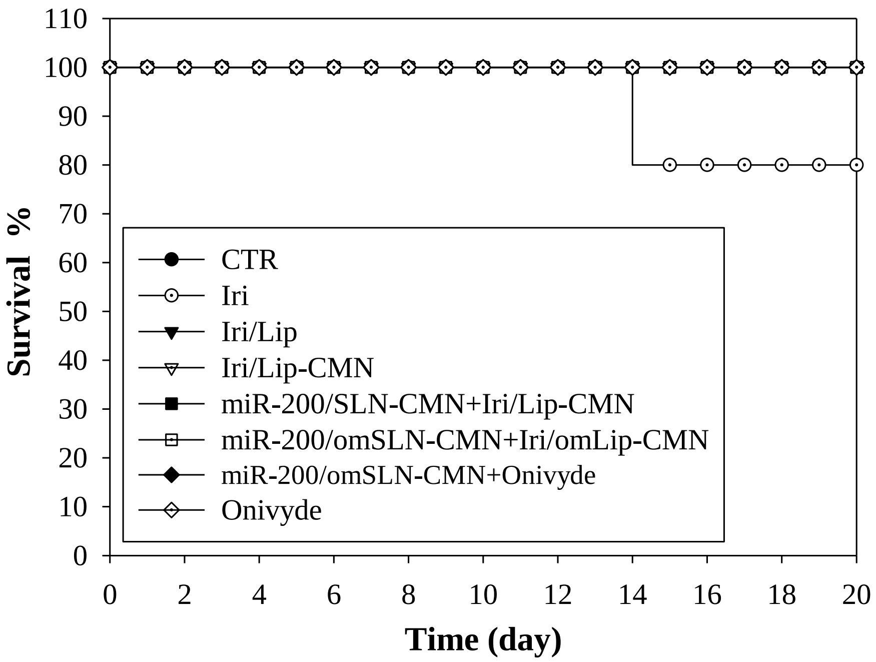

Figure S7. Survival % of SAS -bearing mice treated with different formulations at the final endpoint of the 20th day.
